# Ex Vivo Immuno-Oncology Platform Reveals Spatial T Cell Infiltration Patterns Linked to ATR Inhibition Responses in High-Grade Serous Ovarian Cancer

**DOI:** 10.1158/2326-6066.CIR-25-0743

**Published:** 2026-01-21

**Authors:** Ashwini S Nagaraj, Matilda Salko, Aditi Sirsikar, Ziqi Kang, Erdogan Pekcan Erkan, Elina A Pietilä, Iga Niemiec, Jie Bao, Giovanni Marchi, Angéla Szabó, María Hincapié-Otero, Anastasia Lundgren, Kirsten Nowlan, Sanna Pikkusaari, Anna Kanerva, Johanna Tapper, Riitta Koivisto-Korander, Heini Lassus, Liisa Kauppi, Sampsa Hautaniemi, Anna Vähärautio, Jing Tang, Eliisa Kekäläinen, Ulla-Maija Haltia, Anni Virtanen, Joonas Jukonen, Tuula Salo, Anniina Färkkilä

**Affiliations:** 1 Research Program in Systems Oncology, University of Helsinki, Helsinki, Finland; 2 Faculty of Medicine and Health Technology, Tampere University and TAYS Cancer Centre, Tampere, Finland; 3 Department of Bacteriology and Immunology, University of Helsinki, Helsinki, Finland; 4 Department of Obstetrics and Gynecology, Helsinki University Hospital, Helsinki, Finland; 5 Foundation for the Finnish Cancer Institute, Finland; 6 Translational Immunology Research Program, Faculty of Medicine, University of Helsinki and Helsinki University Hospital, Helsinki, Finland; 7 Department of Pathology, University of Helsinki and HUS Diagnostic Center, Helsinki University Hospital, Helsinki, Finland; 8 Department of Oral and Maxillofacial Diseases, University of Helsinki, Helsinki, Finland; 9 Department of Pathology, HUSLAB, Helsinki University Hospital, Helsinki, Finland; 10 Unit of Population Health, Medical Research Center Oulu, Finland; 11 University of Oulu and Oulu University Hospital, Oulu, Finland; 12 Department of Obstetrics and Gynecology, and Clinical trials unit, Comprehensive Cancer Centre, Helsinki University Hospital, Helsinki, Finland; 13 iCAN digital precision cancer medicine, Helsinki, Finland; 14 Institute for Molecular Medicine Finland, Helsinki Institute of Life Sciences, University of Helsinki, Finland

## Abstract

Identifying new therapeutic approaches in high-grade serous ovarian cancer (HGSC) requires the development of more accurate preclinical models that replicate the patient-specific tumor and its microenvironment. To address this, we established immunocompetent patient-derived cultures (iPDCs) for HGSC, cultured on a physiologically relevant human omentum-gel matrix. We developed a high-throughput platform that combines drug testing, histological analysis, genomic profiling, single-cell studies, and spatial biomarker discovery. Our results from 47 tumors showed that iPDCs recapitulated the tumor genomic and histological characteristics, while also retaining the intratumoral immune cells. The iPDC treatment responses correlated significantly with the patients’ clinical treatment responses. Using iPDCs and scRNAseq, we identified potentially effective therapeutic options for patients with recurrent HGSC linked to distinct tumor cell states and mechanisms of resistance. High-throughput drug response profiling with single cell-imaging identified ataxia telangiectasia and Rad3-related inhibitor (ATRi) combined with an immunotherapy targeting Autotaxin as a promising new combination treatment for HGSC. Using hyperplexed imaging and the spatial analysis, we discovered that ATRi responses were associated with significant increases in both intra- and peritumoral T cell infiltration, particularly in PD1^+^ CD8^+^ T cells. Additionally, the ATRi induced reactivation of CD8^+^ T cells was linked to spatial interactions with replication stress positive tumor cells. Thus, our iPDC platform presents as a representative high-throughput ex vivo model to advance precision oncology in HGSC uncovering ATRi-immunotherapy combination as a potentially effective therapeutic option for clinical translation.

## Introduction

High-grade serous ovarian carcinoma (HGSC) is the most common subtype of ovarian cancer, often diagnosed at an advanced stage with 10-year survival of 15% (1). Nearly 80% of HGSCs metastasize to omentum, a large adipose connective tissue covering stomach and intestine, which provides a metabolically compatible and inflammatory microenvironment for HGSC (2). The standard of care for HGSC includes primary debulking surgery followed by platinum-based chemotherapy. Despite initial treatment response, recurrence often occurs within three years of treatment. Poly (ADP-ribose) polymerase inhibitors (PARPi) have improved patient outcomes when used as a maintenance treatment after first-line chemotherapy, particularly in patients with homologous recombination deficiency (HRD), and/or sensitive to platinum-based chemotherapy (3). However, 35-45% of the patients discontinue the treatment due to resistance to PARPi (4,5), suggesting that novel combination strategies are required to overcome PARPi resistance.

Single-agent immune checkpoint inhibitors (ICIs) have shown limited efficacy in HGSC (6). The lack of success with ICIs in HGSC is likely due to a variety of immunosuppressive cells and mechanisms in the tumor microenvironment (TME) (7). Targeting these mechanisms is one of the strategies to improve the efficacy of ICIs. Towards this, mouse model studies display the importance of enhancing CD8 T cell response via activating interferon signaling in dendritic cells (DCs) or by depleting myeloid-derived suppressor cells (MDSC) (8,9).

To improve the therapeutic benefit of ICIs, their combination with chemotherapy, PARPi, or anti-angiogenic agents are under clinical investigation (10,11). Interestingly, results from the TOPACIO Phase I/II clinical trial showed that a HRD surrogate signature predicts sensitivity to combination of PARPi, niraparib and pembrolizumab (12). In addition, *BRCA1/2*-defective HGSC has been shown to exhibit increased immunosurveillance with more neoantigens, tumor infiltrating lymphocytes, programmed cell death (PD-1) and its ligand (PD-L1) in comparison to HRP tumors (13–15). These findings highlight the potential for immunotherapy combinations for patients with HGSC. Additionally, DNA damage response inhibitors (DDRi) including cell cycle checkpoint inhibitors are being clinically explored (16). While preclinical studies implicate the benefit of combining of DDRi and ICIs (17), investigation of combinations of ATRi with DNA damaging agents or immunotherapies is required for personalized immuno-oncology in ovarian cancer. However, the efficacy of these agents requires validation in immunocompetent models representing patient tumor-specific immune microenvironment prior to clinical investigation.

A variety of patient-derived *ex vivo* models have been developed to model tumor immune microenvironment, and to evaluate response to immunotherapies (18–20). These studies have utilized rodent collagen-based matrices to culture cells or tissue fragments, which may hinder the faithful recapitulation of the functional human HGSC TME. Human tissue-specific extracellular matrices (ECMs) have been produced to model the primary and metastatic TME (21,22). Towards this, Neilson et al., have generated and characterized a human omentum gel matrix (OmGel) from tumor-free omentum, which has the potential utility for studying omental metastatic microenvironment across a plethora of tumor types with frequent metastasis to omentum (23). Here, we established a functional precision immuno-oncology platform using HGSC patient-derived immunocompetent cultures (iPDCs) in patient-derived OmGel matrix. We validated that the iPDCs preserve tumor and immune cell features of the native tumor and recapitulate the clinical responses to chemotherapy and PARPis. By coupling single cell image-based drug responses with immune cell functional states, we show that iPDCs reveal patient-specific vulnerabilities to ATRi combined with immunotherapies. ATRi tumor-specific cytotoxic responses associate with enhanced intratumoral PD1^+^ CD8^+^ T cell infiltration and activation linked to spatially interacting, replication stress-positive tumor cell phenotypes in HGSC.

## Materials and Methods

### Patient recruitment and tissue samples

Tissue samples were collected as a part of ONCOSYS-OVA observational clinical trial including patients with ovarian cancer (NCT06117384) at Helsinki University Hospital (HUH) in accordance with the ethical standards from the 1975 Declaration of Helsinki. Each patient gave their written informed consent, and the use of samples and clinical data was approved by the local Ethics Committee (HUS334/2021). Tissue samples were collected from HGSC patients undergoing primary debulking surgery (PDS) (n=41) or secondary surgery (n=3).

### Tumor tissue processing

Altogether, we processed 47 HGSC tumors from 44 patients and established iPDCs from 37 tumors. Freshly resected tumor tissue was cut into multiple pieces (approximately 100 mm^3^ per piece). Three to six pieces were snap frozen in liquid nitrogen for DNA, and RNA, isolation, one piece was fixed in 10% formalin for histopathology, and t-CycIF analysis. Depending on the availability, 100 mm^3^-2000 mm^3^ cm tumor tissue piece was minced into smaller pieces and digested with 1x Collagenase & Hyaluronidase (# 7912, Stem Cell Technologies), or 1U/mL Dispase II (# D4693, Sigma/Merck), in ADF_+++_ medium (DMEM F12, (# 11320-074 Thermo Scientific) supplemented with GlutaMAX™-I (100X) (#35050-061, Gibco Life Technologies), Penicillin-Streptomycin (10,000 U/ml), (#15140122 Gibco Life Technologies), and HEPES, (# H0887, Sigma)), and 50 U/ mL DNase (# M6101, Promega) at 37 °C for 1 hr rotating incubator. Digested tissue was filtered through 70 μM strainer and centrifuged at 180 g for 10 min at _+_4°C. The pellet was subjected to RBC lysis buffer (# 11814389001, Sigma) for 5 min at room temperature (RT) followed by dilution in AdF_+++_ medium and centrifuged at 180 g for 10 min at _+_4°C. Cells in the resulting final pellet was counted with trypan blue using LUNA-FL™ Dual Fluorescence Cell Counter (# L20001, Logos Biosystems).

### Viability and Immunofluorescence analysis on the dissociated cells

Enzymatically dissociated cells were plated on to Shandon™ Multi-Spot Slides (#9991090, Thermo Fisher Scientific) at a concentration of 30000 cells / 3μL and allowed to air dry at RT. Cells were stained with LIVE/DEAD™ Viability/Cytotoxicity Kit, for mammalian cells (# L3224, Thermo Fisher Scientific), and incubated at 37 °C for 30 min, or cells were fixed with 4% paraformaldehyde for 10 min at RT. Blocking was performed with 2% Triton X-100 + 3 % BSA in 1x PBS for 30 min at room temperature. Cells were stained with anti-Cytokeratin 7 [EPR17078] (Alexa Fluor® 555, ab209601 Abcam, RRID:AB_2728790), and anti-CD45 [HI30] (Alexa Fluor® 647, #304018 BioLegend, RRID:AB_389336) for 1 hr at RT. Images were acquired using Nikon SlideExpress 2 microscope, followed by image analysis using CellProfiler (RRID:SCR_007358). The quantified data was plotted using GraphPad Prism v9 (RRID:SCR_002798).

### Quantitative real-time PCR (RT-qPCR)

RNA was extracted from the cells dissociated with C&H or dispase or from fresh frozen tissues using RNeasy Plus Mini kit (#74134, Qiagen). 500 ng of RNA from each sample was used for cDNA synthesis using High-capacity RNA-to-cDNA kit (#4387406, Thermo Fisher Scientific). Primer sequences for RT-qPCR analysis can be found in STable 1. qPCR mixture was prepared using PowerUp™ SYBR™ Green Master Mix for qPCR (#A25741, Thermo Fisher Scientific), and ran in triplicate technical replicates for each sample using CFX 384 Touch Real-Time PCR Detection System (BioRad). RPS13 was used as a reference for normalization, and data quantified using 2^–ΔΔCT values. Quantified data was plotted using GraphPad Prism v9 (RRID:SCR_002798).

### Establishment of patient-derived cultures

Patient-derived OmGel was prepared as previously described (23). HGSC tumor-dissociated cells were mixed with Cultrex RGF Basement Membrane Extract (BME), Type 2 (# 3533-010-02 R&D Systems), or 2.5 mg/mL OmGel at a concentration of 1000 cells / μL and plated as a droplet in 48-well or 96 well plates. OmGel mixture was prepared as follows: appropriate volume of cell suspension was mixed with, 2.5 mg/mL OmGel, 33.3 ug/mL aprotinin (# A6279 Sigma), 0.3 U thrombin (#T6884 Sigma), and 0.5 μg/mL of fibrinogen (# 341578 Sigma). Addition of fibrinogen initiates solidification of the gel, hence it was added just before plating the suspension. Droplets containing BME or OmGel resuspended cells were allowed to solidify at 37 °C incubator for 1 hr. iPDC culture medium II (STable 2) supplemented with growth factors and cytokines was added on top of the droplets and cultured for 4-10 days depending on the sample.

### Quantification of number of iPDCs per sample

On day 4-10 of the BME or OmGel cultures, bright-field images from 4 wells were taken at 10x magnification, and the number of cell clusters >3000 μm2 was counted using ImageJ-Fiji (RRID: RRID:SCR_002285). The data was plotted using GraphPad Prism v9 (RRID:SCR_002798).

### Whole-exome sequencing

iPDCs were harvested using Cultrex Organoid Harvesting solution (# 3700-100-01 R&D Systems), and DNA was extracted using AllPrep DNA/RNA Mini Kit (80204 Qiagen). 50ng of gDNA from blood (germline, n=11), fresh frozen tumor (n=13), FFPE tumor tissue (n=2), and iPDC (n=10) samples were subjected to WES. WES libraries were prepared using Twist Comprehensive Exome according to the manufacturer’s instructions. This process involved the enrichment of exonic regions, capturing a comprehensive representation of the protein-coding regions of the human genome. Prepared libraries were subjected to high throughput sequencing with Illumina NovaSeq 6000 system using S4 flow cell (Illumina, San Diego, CA, USA) and v1.5 chemistry. DNA samples were subjected to quality control, alignment to the reference human genome, deduplication, estimation of cross-sample contamination, and discovery of genetic variants. We assessed organoid-tissue mutations concordance and Variant allele frequency (VAF) correlation using 10 organoid-tissue pairs. In addition, the pathway analysis from WES data of matched primary and relapse tumors were analyzed using the ConsensusPathDB algorithm (RRID:SCR_002231) (24).

### WES data processing and analysis

Bioinformatic processing of DNA samples involved several steps, including quality control, alignment to the reference human genome, deduplication, cross-sample contamination estimation, and variant discovery. Sequenced DNA reads underwent quality control and trimming steps using FastQC (RRID:SCR_014583) (25) and Trimmomatic tools (RRID:SCR_011848) (26). Subsequently, the high-quality reads were aligned to the reference human genome GRCh38.d1.vd1 using BWA-MEM (RRID:SCR_010910) (27) with default parameters, subjected to deduplication with Picard tool and base quality recalibration using the Genome Analysis Toolkit (GATK) (RRID:SCR_001876) version 4.1.9.0 (28). Cross-sample contamination estimation was conducted using GATK 4.1.9.0, with a contamination estimation threshold established at 15%. Somatic short variants were called through the collective analysis of multiple tumor samples against a singular matched normal for each patient, using GATK 4.1.4.1 Mutect2 according to established best practices. We used a Panel of Normal (PoN) generated with 181 normal samples from the prospective, longitudinal, multiregion observational study DECIDER (Multi-Layer Data to Improve Diagnosis, Predict Therapy Resistance and Suggest Targeted Therapies in HGSOC; NCT04846933) and 99 TCGA normals. A detailed description of PoN creation can be found in Lahtinen et al (29). Subsequently, GATK FilterMutectCalls was used for variant filtration, keeping only those variants that successfully passing all filters. Variant allele frequencies (VAF) were computed based on the read depths for reference and alternate alleles (AD field). We annotated the using ANNOVAR 20191024 (RRID:SCR_012821) (30).

To assess the alignment between iPDC and tissue samples, we calculated tissue-based concordance (Ct) by determining the ratio of shared mutations in both iPDC (i) and tissue samples (t) to mutations found in the tissue (t) Ct = (Mi ∩ Mt) / Mt. Concordance analysis was conducted for iPDC-tissue pairs, with each pair consisting of iPDC and tissue samples derived from the same patient and organ. In total, 10 iPDC-tissue pairs were analyzed. Like iPDC-tissue concordance, we computed organoid-tissue Variant Allele Frequency (VAF) correlation for 10 pairs composed of iPDC and tissue samples from the same patient. Exonic mutations were collected from both primary and relapse samples for patients C865 and C429. The mutated genes identified from these samples were then subjected to over-representation analysis using the ConsensusPathDB algorithm (24).

### Flow cytometry and data analysis

Tumor tissue or iPDCs were dissociated using Dispase II (# D4693, Sigma/Merck), and the cells were stained with two different antibody panels. First immune cell panel for comparison of dissociation methods consisted of the following antibodies: anti-CD14 PE (18D11, #21620144, ImmunoTools, RRID: AB_3251503), anti-HLA-Dr PerCP-CY5.5 (G46-6, #552764 BD, RRID:AB_394453), anti-CD8 PE-Cy7 (RPA-T8, #557746, BD, RRID:AB_396852), anti-CD4 APC (MEM-241 #21270046, ImmunoTools, RRID: AB_3718639), anti-CD56 AF700 (B159, #557919 BD, RRID:AB_396940), anti-EpCAM APC/Fire 750 (9C4, #324233 BioLegend, RRID: AB_2629702), anti-CD45 BV421 (HI30, #563880 BD, RRID: AB_2744402), anti-CD16 BV510 (3G8, #563829 BD, RRID:AB_2744296), anti-CD19 BV605 (HIB19, #302243 BD, RRID:AB_2562014), anti-CD11c, BV11c (B-ly6, #563130 Fisher Scientific, RRID:AB_2738019), anti-CD3 BV786 (CD3 SK7, #563800 BD, RRID: AB_2744384), dead cell marker (DCM) FITC (#L2301,Thermo Fisher Scientific). Second panel for the analysis of the functional states of the immune cells: anti-CD56 FITC (MEM-188, #21270563 ImmunoTools, RRID: AB_3251502), anti-CD4 PE-CF594 (RPA-T4, #562281 BD, RRID: AB_11154597), anti-Ki67 PE-CY7 (B56, #561283 BD, RRID: AB_10716060), anti-Granzyme B APC (GB11, #GRB05 Thermo Fisher Scientific, RRID: AB_2536539), anti-IFN γ AF700 (B27, #557995 BD, RRID: AB_396977), DCM BV510 (#L34965 Thermo Fisher Scientific), and the remaining antibodies were the same as in the immune cell profiling panel. Samples were run using LSRFortessa™ Cell Analyzer (BD). From the flow cytometry data, DCMneg and EpCAM neg cells were gated (STable 3) using FlowJo v10 (RRID:SCR_008520) and exported as csv files for further analysis using CYTO (31). The proportions of the cell types from the clusters generated in CYTO were plotted using R-script. For the analysis of functional states of immune cells following treatment with olaparib or pembrolizumab, DCM^-^ EpCAM^-^ cells were imported into CYTO and clustered into 25 clusters using FlowSOM (RRID:SCR_016899). Clusters were annotated into CD8^+^ T cells, CD4^+^ T cells, CD56^+^ NK cells, or CD11c^+^ myeloid cells based on the z-score of the cell type-specific markers per cluster. The annotated clusters were analyzed for log2FC in the mean intensity of GrzB, IFNγ, Ki67, and HLA-Dr per individual cell type in each treatment normalized to control.

The cell type annotation was performed using TRIBUS (RRID:SCR_027367) (32). Outliers were removed using the 99.9th percentile for input marker intensity, normalized with arcsin transformation with a cofactor of 5, and scaled by Z-score. TRIBUS was run with the following parameter settings of sigma=1, learning_rate=1, clustering_threshold=10, undefined_threshold=0.0005, and other_threshold=0.3. A total of 5 cell types were annotated, including NK cells (CD56^+^), Monocytes (CD14^+^), Myeloids (CD11c^+^), T cells (CD3^+^ CD8^-^), and T cells (CD3^+^ CD8^+^).

### Drug treatment and immunofluorescence staining

Dissociated patient-derived cells were embedded as 1000 cells/μL in 25μL of OmGel matrix on a CellCarrier 384 Ultra microplates (PerkinElmer), or in 10μL of OmGel matrix on a Cell Culture 96 well F-bottom microplate (Greiner). Drops were left to solidify for 1 hr at +37°C before adding iPDC-growth medium. Next day, cultures were treated with olaparib (PARPi, AZD2281; SelleckChem), pembrolizumab (anti-PD-1, Selleckchem), ziritaxestat (ATXi, GLPG1690; MedChemExpress), AMG PERK44 (PERKi; MedChemExpress), Adavosertib (WEE1i, MK-1775; SelleckChem) or berzosertib (ATRi, VE-822; SelleckChem) as single agents (STable 4) or as combinations (STable 5) using TECAN D300e Digital Dispenser (TECAN) in a randomized manner. Dimethyl sulfoxide (DMSO) was used as a control. Chemo- and PARPi-resistant patient-derived iPDCs were cultured on a Shandon Multi-Spot slides (#9991090, ThermoScientific) and treated with cisplatin (S1166; SelleckChem) or Olaparib, WEE1i or ATRi. Drug treatment was carried out for 3-6 days, depending on the growth of iPDCs, as evaluated by phase-contrast microscopy. At the end of drug treatment, cells were stained with live-or-dead stain (#32004, Biotium) following manufacturer’s protocol before fixing with 4% paraformaldehyde for 1 hr at RT. Wells were washed 3x with 1x PBS followed by permeabilization and blocking with freshly prepared 3% bovine serum albumin and 0.3% Triton X-100 in 1x PBS for 1 hr at RT. After removing the blocking buffer, cells were incubated with anti-Cytokeratin 7 [EPR17078] (Alexa Fluor® 555 conjugated, ab209601 Abcam, RRID:AB_2728790), anti-CD45 [HI30] (Alexa Fluor® 647 conjugated, #304018 BioLegend, RRID:AB_389336) and Hoechst (#3342, Thermo Fisher Scientific) in the blocking buffer overnight at _+_4°C. Next day wells were carefully washed 3 times with 1x PBS to remove all the Triton X-100 and unbound antibodies. Wells were left with 50 μL of PBS for imaging.

### High-content single-cell imaging and data analysis

Images of the drug-treated samples (10-17 images per well) were acquired using Opera Phenix High Content Screening System (PerkinElmer) with 40x objective and Andor Zyla sCMOS camera, and processed with Harmony High Content Imaging and Analysis Software (RRID:SCR_023543) (PerkinElmer). Raw image processing was done with BIAS (Biology Image Analysis Software) (Single-Cell Technologies). Cell segmentation and quantification of tumor (CK7^+^), immune cells (CD45^+^), and dead cells (DCM^+^), was performed using CellProfiler (RRID:SCR_007358). Sample-specific threshold was set for CK7 integrated intensity values from the membrane, CD45 mean intensity values from the membrane, and mean intensity values from the nucleus of DCM per sample for further analyses.

The data from CellProfiler was graphically represented as the abundance of live tumor (DCM^-^, CK7^+^) and live immune cells (DCM^-^, CD45^+^) per treatment and per patient sample using RStudio (RRID:SCR_000432). Outlier images which contained fewer than five cells, no CK7^+^ cells, and no CD45^+^ cells were filtered out. Images which contained an excessive number of cells, greater than three standard deviations above the mean, were also excluded. The data was visualized using a ratio of CK7 alive over all alive cells (tumor cell abundance), CD45 positive alive cells over all cells (immune cell abundance). The median tumor and immune abundance proportions per treatment were normalized by the control and the log2 fold change was plotted as the heatmap.

### Single-cell RNA-sequencing and data analysis

Dispase-dissociated single cell suspensions were washed in 1X PBS + 0.04% BSA solution and cell viability was assessed on an automated cell counter. scRNA-seq libraries were prepared with Chromium NextGEM Single Cell 3’ reagent kit (PN-1000128 10x Genomics) and sequenced on an Illumina NovaSeq 6000 instrument at the Sequencing Unit of Institute of Molecular Medicine Finland, using one biological and technical replicate per sample. scRNA-seq data preprocessing was done as described previously (33), QC metrics are listed in STable 6. Briefly, the FASTQ files were processed with the Cell Ranger pipeline v.6.0.1 (RRID:SCR_017344) (10x Genomics) to perform sample demultiplexing, alignment, barcode processing, and UMI quantification. The reference index was built upon the GRCh38.d1.vd1 reference genome with GENCODE v25 (RRID:SCR_014966) annotation. The count matrices of four samples were loaded into Seurat (RRID:SCR_007322) R package (v.4.3.0) and merged, and the miQC algorithm (RRID:SCR_022697) (34) was used for quality control. Cells were assigned to the low-resolution cell types (epithelial tumor cells, stromal cells, and immune cells) based on the expression of marker genes as described previously (33). To assign high-resolution immune cell types, the celltypist algorithm (RRID:SCR_024893) was used with the model “Immune_All_Low”.

The decoupleR R package (v.2.7.1) (RRID:SCR_027127) (35) was used to infer biological activities of 14 signaling pathways available in PROGENy (36). For each sample, pathway activities across cell types were inferred by running the *run_ulm* function with default parameters, and aggregated pathway activities were visualized as a heatmap.

### t-CycIF staining and image analysis

Formalin-fixed paraffin-embedded (FFPE) tumor tissue sections from 12 PDS patient samples were sequentially stained with validated antibodies (STable 7) and scanned using CyteFinder II (RareCyte) as outlined in the t-CycIF protocol (37). Images from each cycle of staining were stitched and registered to create one high-plex image using the ASHLAR algorithm (RRID:SCR_016266) (38). Nuclear segmentation was achieved with the StarDist method by identifying positive nuclear Hoechst staining (39). The mean fluorescence intensity for all markers expressed within each cell was quantified using an in-house python script. Quality control was carried out in CyLinter (RRID:SCR_021157) (https://labsyspharm.github.io/cylinter/) to remove data affected by staining and image processing artifacts, including folds in the tissue, antibody aggregates and shifted cells. To mitigate batch effects and enable data comparison, the raw intensity values from each sample was subjected to a log2 transformation followed by z-score normalization, the mean intensity of normalized pRPA32/RPA2(Ser8) in PAX8^+^ and or CK7^+^ tumor cells per sample were plotted.

The spatial analysis of t-CycIF images was performed by an in-house package (https://github.com/farkkilab/Nagaraj-et-al). To define whether the immune cells resided in the tumor or stroma region, we calculated the proportion of cell types within a radius of 65μm (200 pixels). If > 50% of the detected cells were cancer cells, the immune cell was labeled as tumor-resident; otherwise, stroma-resident. The immune infiltration index was defined as immune density (tumor) divided by immune density (stroma), where the immune density in each region was calculated by the immune cell count in each region divided by the total number of tumor/stroma cells. The tumor stroma interface (TSI) was defined using KDTree-based nearest neighbor search. For each tumor cell, we detected the neighboring cells from the stroma located within a radius of 13 μm (40 pixels), and vice versa. For the immune-TSI distance analysis, we calculated the nearest distance from each immune cell to the TSI cells. The median distance of each immune cell type per slide was then calculated for statistical tests. Log2 fold change for TSI proximity analysis were calculated based on the group-wise means of the spatial metrics (response: Yes/No). When testing for the spatial co-localization of functional markers, we calculated the mean marker intensity within a radius of 65μm (200 pixels) for each tumor cell, after which we calculated the median value per slide. For the analysis of GrzB expression of PD-1^+^ CD8^+^ T cells, we used a pRPA32^+^ or ^-^ tumor cell as the center cell and calculated the mean of GrzB expression of all PD-1^+^ CD8^+^ T cells within a radius to form each data point. The selection of radii were based on the cell-cell distance distribution of the t-CycIF dataset.

### Statistical analysis

Paired Wilcoxon signed-rank test was applied for comparison of organoid numbers between BME and OmGel. Pearson correlation was employed for correlation analysis of the WES data. A p-value threshold of 0.05 was set to determine significant statistical comparisons. Mann-Whitney U test was applied for quantified data from PDC tumor cell response to olaparib treatment in HRD vs HRP PDCs. Pearson’s correlation coefficient was applied to calculate the significance between patient’s PFS and PDC response to Olaparib. Wilcoxon signed-rank test was applied for calculating the significance between duration of PARPi treatments in patient vs PDC response to olaparib. Paired Wilcoxon signed-rank test was used for comparison of immune cell proportions between C&H vs dispase, BME vs OmGel, and tumor vs iPDCs. Wilcoxon signed-rank test was used to compare the proportions of CK7^+^ tumor or CD45^+^ immune cells in each treatment against the control. Statistical significance for TSI proximity analysis was assessed using the Wilcoxon signed-rank test. One-sided Mann–Whitney U test was used for immune-TSI distance analysis. Statistical significance for GrzB and PD-1 expression analyses, and for GrzB expression analysis of PD-1^+^ CD8 T^+^ cells were determined using the Mann–Whitney U test. Statistical significances are denoted as follows: p < 0.05*, p < 0.01**, p < 0.001***, p < 0.0001****.

## Results

### Humanized OmGel matrix significantly improves the growth of HGSC patient-derived cultures

**Fig 1:**
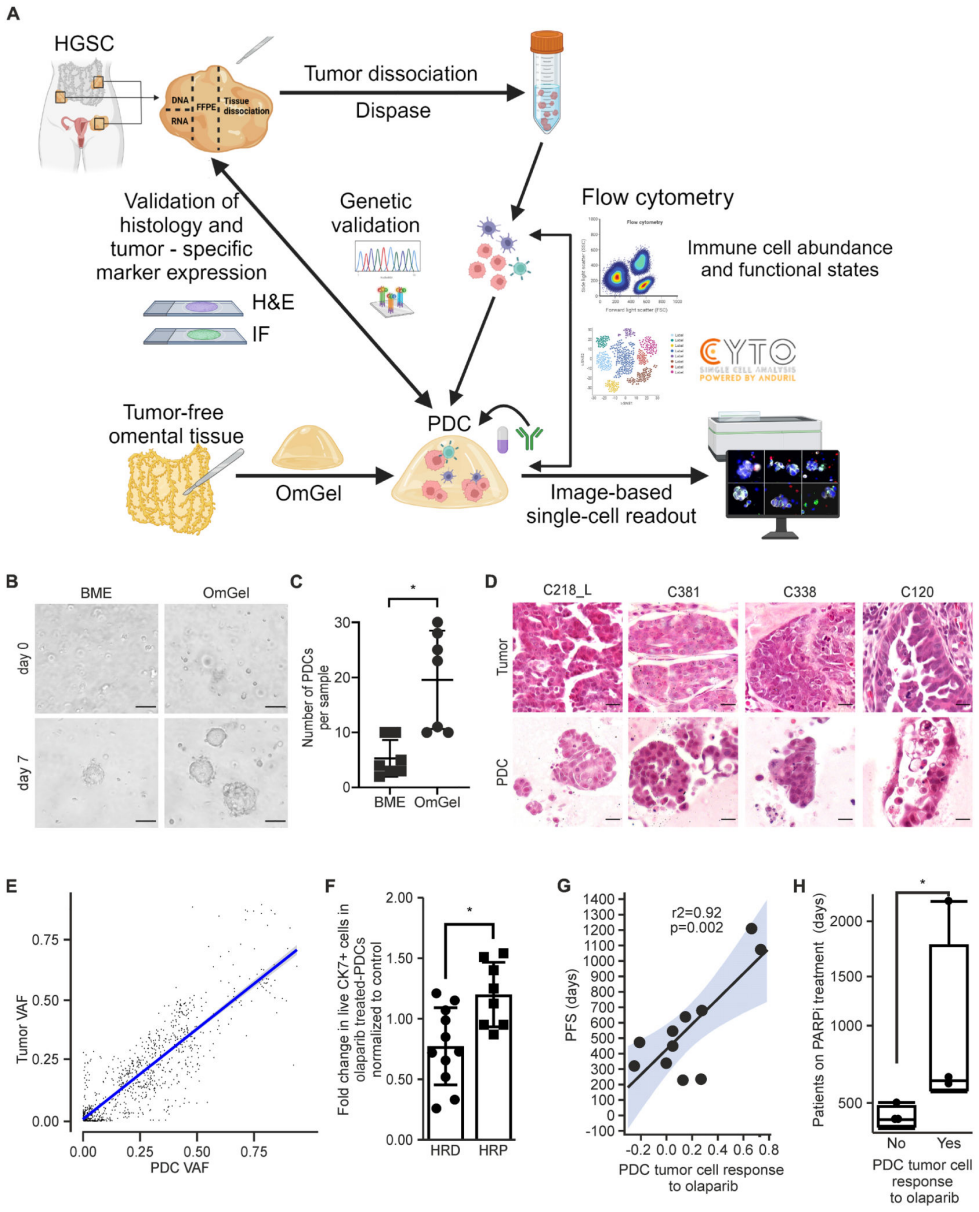
OmGel matrix enables improved growth of HGSC iPDCs that retain source tumor-specific features and immune cells, enabling the investigation of immune cell-specific treatment responses. **A)**. Schematic showing the establishment and validation of iPDCs cultivated in OmGel, followed by drug testing and drug response analysis using image-based single-cell readouts. Created in BioRender. Nagaraj, A. (2025) https://BioRender.com/zszhgmx. **B)**. Bright field images showing HGSC patient-derived cells cultured in basement membrane matrix (BME) or OmGel. Scale bar 50μM. **C)**. Quantification of PDC numbers cultured in BME or OmGel for 4-10 days. Each square/circle represents an individual sample. **D)**. H&E images of tumor tissue and matched PDCs cultured in OmGel for 4-6 days. Scale bar 100μM. **E)**. Scatter plot depicting the correlation of variant allele frequencies (VAF) between tumor and PDCs. **F)**. Quantification of the single-cell imaging data from HRD/HRP PDCs, treated with 20μM olaparib for 4-10 days. Each dot represents an individual PDC. **G)**. Scatter plot depicting the decrease in live CK7^+^ tumor cells from HRD-PDCs upon olaparib treatment and corresponding patient progression-free survival (PFS). **H)**. Box plot showing the tumor cell-specific olaparib response from PDCs vs duration of PARPi treatment in the corresponding patients. Error bars represent standard deviation (SD).

To enable faithful recapitulation of the TME in patient-derived cultures (PDCs), we first established an optimized tissue dissociation protocol via comparing two commonly used tissue dissociation enzymes for adjacent tissue regions: Collagenase/Hyaluronidase (C&H), and dispase for the final yield, viability, abundance of tumor and immune cells, and expression of stress response genes, to optimize tumor- and immune cell yield from the source tissue (SFig 1A). Our results showed no significant difference in the yield or viability between C&H and dispase dissociation (SFig 1B-D). Similarly, the abundance of CK7^+^ tumor cells or CD45^+^ immune cells were not affected by dissociation enzyme choice (SFig 1E,F). However, the expressions of stress response genes *ATF3, FOS*, and *HSPA1* were significantly increased in C&H-dissociated cells compared to dispase-dissociated cells (SFig 1G). The upregulation of stress response genes has been linked to chemoresistance (33,40), and therefore can distort drug response analysis from cancer cells, and could lead to altered immune cell phenotypes. Hence, we continued with dispase dissociation for establishing the PDCs (Fig 1A). To assess the effect of the ECM on the growth of the PDCs, dissociated cells from HGSC primary or omental metastatic tumors were cultured in basement membrane extract (BME) or HGSC patient-derived OmGel for up to one week. Compared to cells cultured in BME, OmGel cultured cells showed significantly improved growth as measured by the number of PDCs with size >3000 μm^2^ (Fig 1B,C; SFig 1H). OmGel cultured PDCs maintained source tumor morphology and expression of tumor-specific marker PAX8 (Fig 1D; SFig 1I). In addition, whole exome sequencing (WES) analysis of tumor and matched PDCs (n=10) showed a strong correlation (r=0.86) of the variant allele frequencies (VAF), and a high median concordance (95.8%) for tumor-specific mutations between tumor and PDC pairs (Fig 1E; SFig 1J).

### Optimized PDCs recapitulate clinical HRD genotype-specific clinical treatment responses to PARP inhibitors

Clinically, PARP inhibitor maintenance therapy is effective particularly in HGSC patients with HRD and or *BRCA* mutation (41). However, nearly 30% of the patients with a germline *BRCA* mutation relapse within the first 2 years of PARPi maintenance therapy (42). We next tested if the PDCs established from treatment-naive patient-derived tumors exhibit HRD genotype-specific responses to PARPi. The homologous recombination (HR) status of all tumor samples used in this study was assessed with the genomic ovaHRDscar algorithm (43). We treated 19 treatment-naïve HRD (n=11) and HRP (n=8) PDCs with olaparib for up to one week and evaluated the responses using an optimized single-cell imaging-based readout (Fig 1A). The result showed that the tumor cells from HRD PDCs responded significantly better as compared to the HRP PDCs (SFig 1K; Fig 1F). Furthermore, PDC tumor cell responses to olaparib significantly correlated with the patient’s progression-free survival (PFS) as well as the duration of PARPi treatment in these patients (Fig 1G,H). This significant correlation of PFS and PARPi maintenance treatment (44) led us to focus on clinical correlations with PARPi responses, rather than with chemotherapy responses. In summary, we show the successful generation of HGSC PDCs that faithfully recapitulate the source tumors, and patient clinical treatment responses to PARPi.

### PDCs preserve the source tumor immune cells and exhibit cell-type specific phenotypic responses to immunotherapies

**Fig 2:**
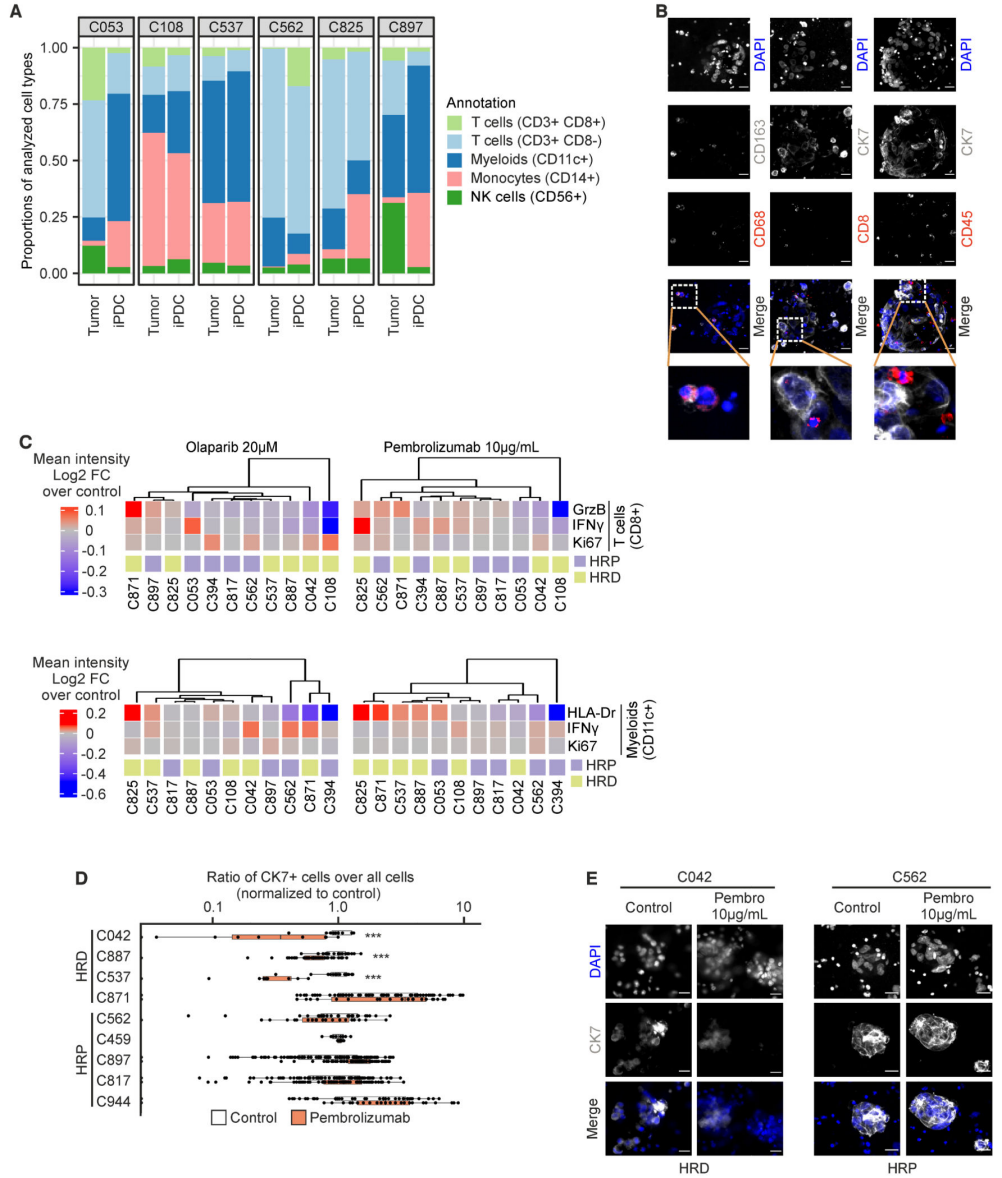
Source tumor immune cells are preserved by PDCs and the immunotherapy responses of PDCs occur in a cell-type specific manner. **A)**. Bar plot showing the proportions of indicated immune cells in tumor and matched iPDCs. iPDC cell proportions displayed originate from viable cells at the experiment endpoint, and are annotated from the clusters shown in SFig 2B. **B)**. Representative IF images of day 4 iPDCs stained with indicated antibodies. Scale bar 50μM. **C)**. Heatmap showing flow cytometry analysis of HLA-Dr, IFNγ, and Ki67 expression in CD11c^+^ myeloid cells or GrzB, IFNγ, and Ki67 expression in CD8^+^ T cells, from iPDCs upon treatment with 20μM olaparib or 10mg/mL pembrolizumab for 3-6 days as Log2FC mean intensities normalized to control. **D)**. Box plot showing the ratio of CK7^+^ cells over all cells normalized to control 4-6 days post pembrolizumab treatment. Each dot represents an individual image. **E)**. Representative IF images of control or pembrolizumab-treated iPDCs stained with anti-CK7. Scale bar 50μM.

To evaluate if the dissociation method or culture matrix influences the recovery of immune cell types, we performed multi-parameter flow cytometry on C&H or dispase-dissociated cells or cells cultured in BME or OmGel. The live non-tumor cell populations (DCM^-^ EpCAM^-^) were gated in FlowJO and further annotated to distinct immune cell populations using our single-cell analysis tool CYTO based on FlowSOM (SFig 2A,B) (31). The immune cell proportions did not show significant differences between dissociation or culture matrixes, indicating a robust recovery of the immune cell populations (SFig 2C,D). Moreover, the immune cell fractions displayed patient-specific profiles, without statistically significant differences between the source tumor and matched PDCs cultured for 3–7-days (Fig 2A, SFig 2E). T cells, NK cells and myeloid cells required for eliciting response to immunotherapies were faithfully maintained in the cultures (Fig 2A). Additionally, immunofluorescence (IF) staining highlighted not only the presence of CD45^+^ immune cells, CD8^+^ T cells, CD68^+^ and CD163^+^ macrophages in the cultures, but also that the immune cells were spatially interacting with the CK7^+^ tumor cells (Fig 2B).

We next assessed the immune cell functional responses to PARPi or immune checkpoint blockade in 11 PDCs (SFig 2F). We observed patient-specific immune cell activation indicated by an increase in HLA-Dr, IFNγ, or Ki67 in CD11c^+^ myeloid cells, or GrzB, IFNγ, or Ki67 in T cells. Notably, pembrolizumab treatment increased antigen presentation of myeloid cells predominantly in HRD iPDCs (4/6 HRD iPDCs vs 1/5 HRP iPDCs), while CD8^+^ T cell activation was independent of the tumor HRD status (Fig 2C). Next, we investigated tumor cell responses in iPDCs upon pembrolizumab treatment via single cell image-analysis. Interestingly we observed that 3/4 HRD iPDCs, but none of the HRP-iPDCs responded to pembrolizumab treatment (Fig 2D,E), in line with the clinical observation that HRD tumors can be more responsive to ICB (12). In summary, these results show that our immune competent PDCs (iPDCs) preserve the functional immune cells from the source tumor, and enable functional testing of patient-specific responses to immunotherapies in HGSC.

### Investigation of PARPi or chemotherapy resistant tumors reveal drug sensitivities linked to distinct transcriptomic programs

**Fig 3:**
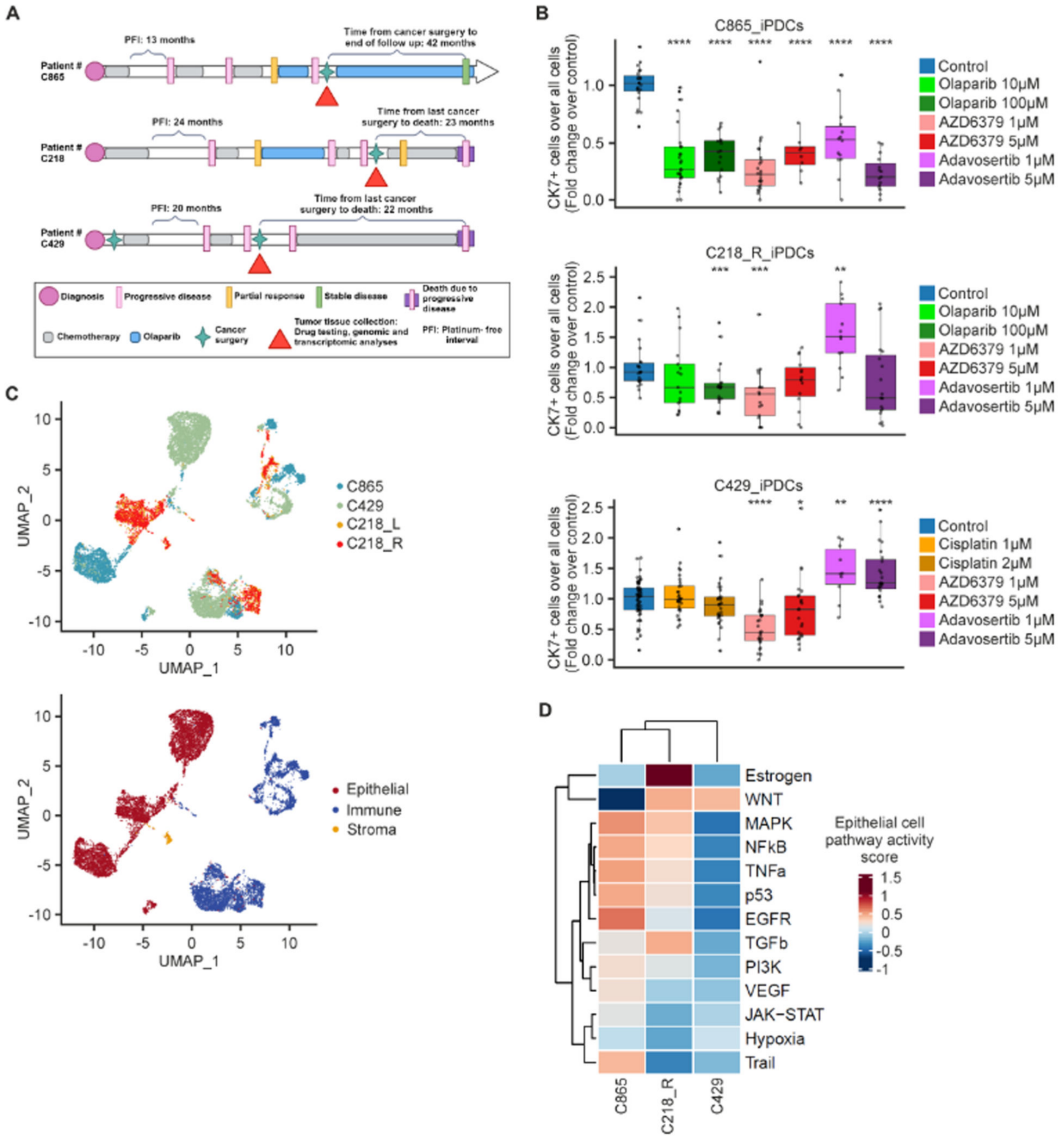
iPDCs recapitulate clinical response to olaparib or chemotherapy and reveal new drug sensitivities. **A)**. Schematic representation of clinical course of three patients who were resistant to PARPi or chemotherapy at the time of tumor tissue collection for scRNA seq, and drug testing using iPDCs. Created in BioRender. Nagaraj, A. (2025) https://BioRender.com/l8sfddw. **B)**. Box plots showing the ratio of CK7^+^ cells over all cells, normalized to control upon 3-4 days of indicated drug treatments on the iPDCs of the patients shown in (A). Each dot represents an individual image. **C)**. UMAP plot of all cells passing the quality control from scRNAseq data, colored by patient code (top), or by the cell types (bottom) **D)**. Heatmap of PROGENy scores for epithelial cells from the samples shown in (C).

To assess whether our iPDC model could be used to study new therapeutic options for recurrence-stage cases that have exhausted standard therapies, we processed recurrence tumors from three patients whose disease had progressed during olaparib or chemotherapy, and subjected the tumors for genomic and single-cell transcriptomic analyses, and drug testing on the iPDCs (Fig 3A). In all three patients, sampling was performed in the secondary surgery in case of oligometastatic recurrent disease. While two patients (C865, C218) had received chemotherapy and PARPi olaparib before the surgery, the third patient (C429) was treated with chemotherapy. Interestingly, patient C865 has continued olaparib maintenance therapy after the surgery, and she is still showing a response with a stable disease (42 months). Conversely, the other two patients, C218 and C429, developed progressive disease after the surgery leading to death.

The recurrence tumors collected from three patients (Fig 3A) were processed for WES, scRNA-seq, and iPDC establishment for drug response profiling. The iPDCs from the two patients treated with chemotherapy and olaparib (C865, C218_R (right ovary)) were treated with AZD6379 (ATRi), adavosertib (WEE1i), or olaparib (PARPi), while the iPDCs from the chemotherapy resistant patient (C429) were treated with AZD6379, adavosertib, or cisplatin (Fig 3B). Our results showed that olaparib, AZD6379, and adavosertib treatment resulted in a significant reduction in CK7^+^ tumor cells in the iPDCs from olaparib-responder patient (C865) compared to the control. Consistent with clinical responses in the olaparib (C218_R), or chemotherapy (C429) resistant patients, no significant reduction in CK7^+^ cells were detected following treatment with 10 uM olaparib or cisplatin in iPDCs from, respectively. Clinically, this patient was also able to continue olaparib for >42 months after the secondary surgery. The iPDCs from the two resistant patients showed significant responses to ATRi, but not to WEE1i, identifying these as potential treatment options for these patients. Together, these results show a proof of concept that iPDCs recapitulate patient-specific clinical responses to olaparib or chemotherapy and revealed ATR inhibition as a potential new treatment option for two of the therapy-resistant patients, taking into account previous studies also presenting ATRi as a potential therapy option for replication stress-affected HGSC (45).

To gain deeper insights into the molecular features driving drug sensitivity and resistance we performed scRNA-seq and WES on tumors from the aforementioned patients, including an additional left ovary sample from C218 (C218_L) in order to assess the level of homogeneity between tumor sample sites. Uniform Manifold Approximation and Projection (UMAP) plots showed patient-specific as well as cell type-specific clusters (Fig 3C; SFig 3A). In addition, epithelial cells and macrophages were the predominant cell types accounting for >70% of all cells identified with scRNA-seq (SFig 3B). Epithelial cell progeny pathway scores revealed enrichment of estrogen, WNT, and TGFb pathways implicated in resistance to PARPi (46–48) in the olaparib resistant tumor (C218_R), while the chemotherapy resistant tumor (C429) was enriched only in WNT signaling (SFig 3C). On the contrary, TRAIL (tumor-necrosis factor related apoptosis-inducing ligand) pathway was enriched in the tumor from the olaparib sensitive patient (Fig 3D). Furthermore, cytotoxic T cells and macrophages from resistant tumors showed distinct activities related to WNT, and JAK-STAT signaling, respectively (SFig 3D,E). Further, WES data from two patients with available matched primary and resistant samples (C865, and C429), showed no enrichments in mutations in TNF, EGFR, PI3K or WNT pathways suggesting that the resistance associated pathways were driven mostly by cancer cell state dynamics.

Multiple mechanisms contribute to PARPi resistance, including restoration of the HR, or BRCA1/2 protein expression (49). Consistent with our observation on olaparib resistance in C218, mutation analysis revealed a potential somatic reversion of a previously detected germline alteration (*BRCA1:c.737delT*): in both in tumor and iPDC WES data we observed a deletion spanning from intron 6 to 7 to the middle of exon 10 on chromosome 17 (chr17:g.43092980-43102933del). In line with this, a functional HRD test based on RAD51 foci (50) showed functional restoration of HR, while the samples from the other two patients were found to be HRD. In addition, we observed a transcriptional enrichment of proliferation-related pathways such as, G2M Checkpoint, E2F Targets, MYC Targets in the cancer cells, previously associated with PARPi resistance (49). Collectively, our data supports the alignment of the iPDC functional responses to the tumor molecular profiles and resistance mechanisms, which can aid in identifying new therapeutic options for patients with recurrent HGSC.

### iPDC platform identifies responders to ATRi and immunotherapy combinations

**Fig 4:**
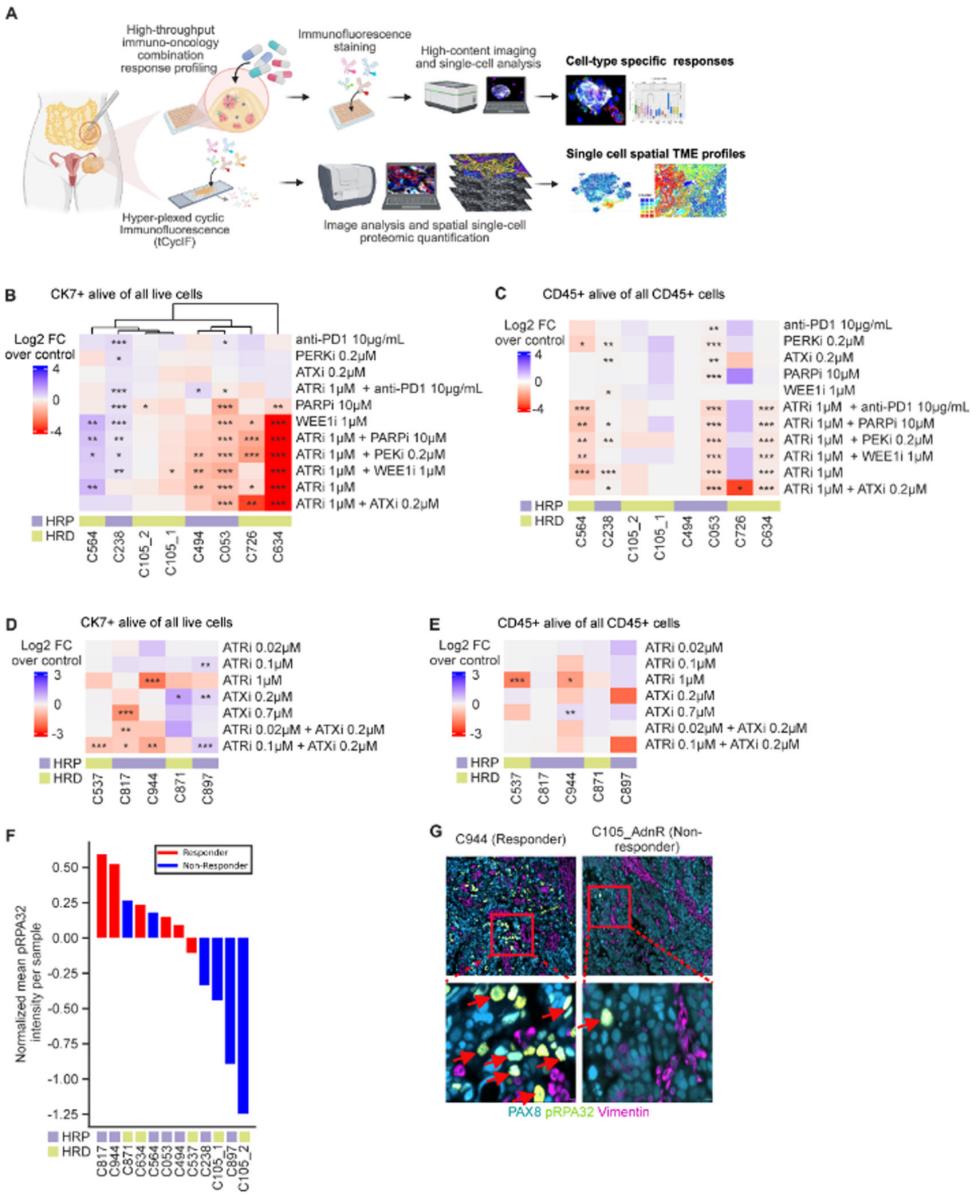
The iPDC platform enables testing of immuno-oncology agents and evaluation of tumor-specific responses to treatments. **A)**. Schematic showing the workflow for testing immune-oncology agents in 384-well format followed by analysis of tumor and immune cell-specific responses using high-content single cell imaging and t-CycIF. Created in BioRender. Jukonen, J. (2025) https://BioRender.com/zeq575b. **B)**. An unsupervised hierarchical clustering showing the Log2FC in live CK7^+^ tumor cells over all live cells in each treatment condition normalized to control. **C)**. Heatmap showing the Log2FC in live CD45^+^ immune over all CD45^+^ cells in each treatment condition normalized to control. Drug treatments were performed for 4-6 days. **D)**. Heatmap showing the Log2FC in live cells normalized to control. **E)**. Heatmap showing the Log2FC in the live CD45^+^ immune over all CD45^+^ cells normalized to control. **F)**. Waterfall plot depicting quantification of the mean intensity of pRPA32 in PAX8^+^ tumor cells normalized per sample. In the patient code, _1 is Omentum, and _2 is Right Adnex. **G)**. Representative t-CycIF images on FFPE sections of the tumors from responder (C944) and non-responder (C105_A) to the ATRi combinations, with indicated antibodies. Red arrows point towards pRPA32-RPA2 (Ser8)^+^ and PAX8^+^ cells. Scale bar, 150μM for the top and 25μM for the bottom panel.

As we show the iPDC models treatment responses to recapitulate clinical ones, we established a high-throughput pipeline for testing iPDCs for combinatorial drug treatments (Fig 4A). We subjected the iPDCs for testing of a total of 54 different mono- and combination therapy conditions of DNA damaging and immunotherapies using an automated drug dispenser (STable 4,5). The agents for drug testing were chosen based on prior clinical and preclinical studies, including a Phase II clinical trial, and a syngeneic ovarian cancer mouse model study (9,43,47,49), suggesting ATRi, and inhibitors of the LPA pathway as promising therapeutic agents. To enable cell-type specific response readouts, we employed a four channel IF staining followed by high-content imaging to measure single-cell cytotoxic responses. For concurrent tumor sample biomarker discovery, the same tumor was subjected to single-cell spatial analysis using hyperplexed imaging.

Unsupervised hierarchical clustering of eight iPDCS for live CK7^+^ tumor cells showed that the samples clustered based on the drug treatment responses (SFig 4A). Statistical comparison revealed that ATRi single and combinatorial treatments caused significant tumor cell death in a sample-specific manner in 4/8 iPDCs (Fig 4B), independent of the tumor HR status. However, 50% samples responding to ATRi also exhibited a decrease in live CD45^+^ immune cells indicating toxicity to immune cells (Fig 4C). We next tested if lowering the concentrations of ATRi could yield tumor-cell specific cytotoxic effects. We selected ATXi for further validation and functional analysis in combination with ATRi as this agent is known to induce CD8^+^ T cell activation via inducing IFN-signaling in cancer cells (9). In iPDCs established from 6 new HGSC patients, we observed that 3/5 iPDCs were sensitive to the lower dose combination of ATRi with ATXi without causing a significant reduction in live immune cells (Fig 4D,E). This sensitivity was associated with an increased CD8^+^ T cell activation indicated by higher GrzB and IFNγ, along with CD11c_+_ myeloid cell activation with increased IFNγ in 3/4 of the sensitive iPDCs (SFig 4B). These results confirm the efficacy and immune activation of the ATRi treatment in HGSC, highlighting the added value of our iPDC platform in identifying tumor cell-specific cytotoxic responses and also immune cell functional states during new single-agent and combination therapies.

### Tumor - CD8^+^ T cell spatial and functional crosstalk underlies responses to ATRi

**Fig 5:**
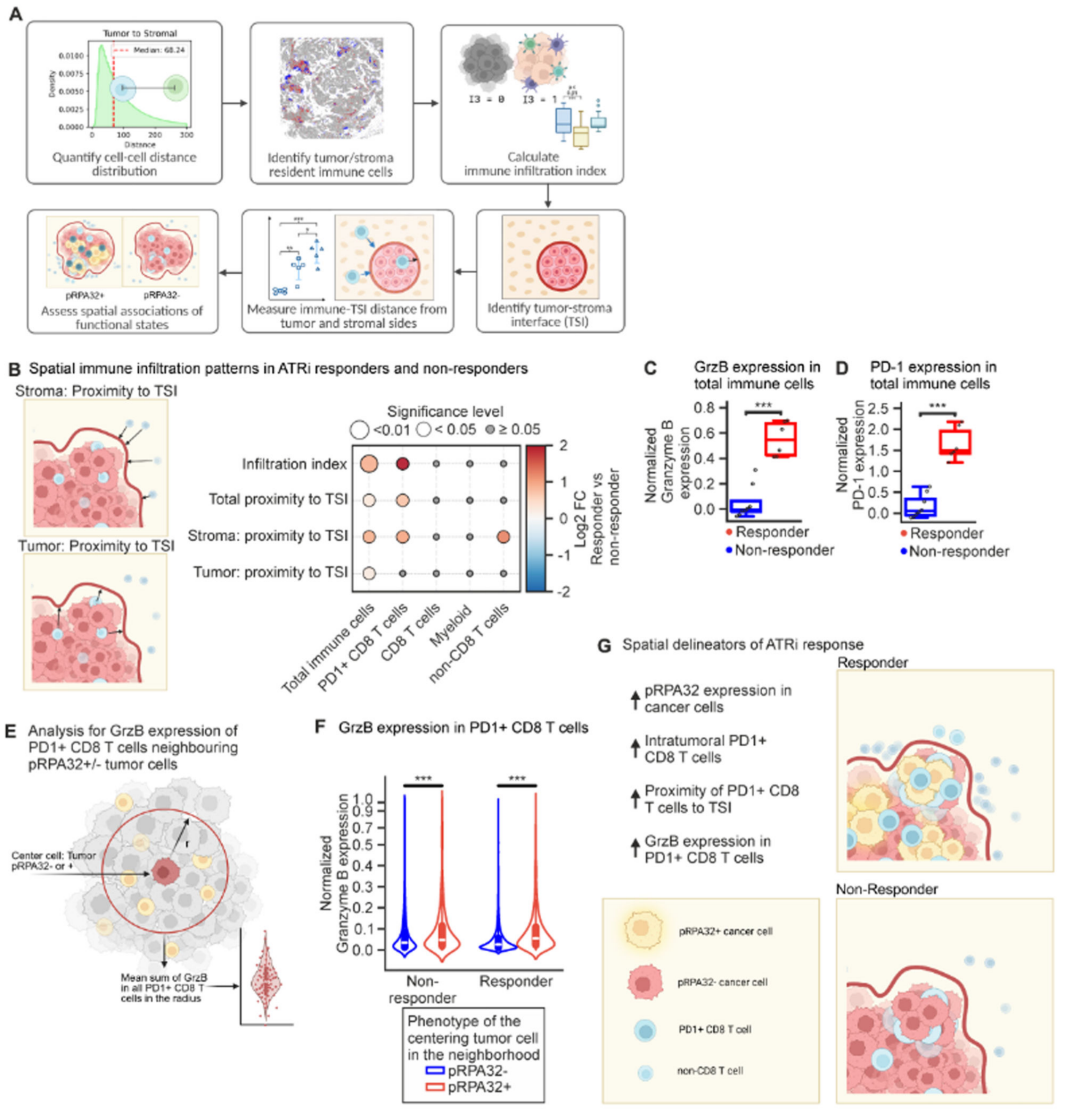
ATRi responses are underlined by cancer - T cell spatial and functional crosstalk. **A)**. Schematic for spatial analysis of t-CycIF images, enabling the quantification of tumor immune infiltration patterns. Created in BioRender. Kang, Z. (2025) https://BioRender.com/crafm3c. **B)**. Graphical example of the spatial TSI proximity analysis and heatmap summarizing spatial analysis results. Graphical components created in BioRender. Kang, Z. (2025) https://BioRender.com/oatziln. **C)**. Box plot showing Granzyme B (GrzB) expression in immune cells adjacent to tumor cells between responder groups. Each data point in the box plots represents the median value per sample. **D)**. Box plot showing PD-1 expression in immune cells adjacent to tumor cells between responder groups. Each data point in the box plots represents the median value per sample. **E)**. A graphical example showing the spatial analysis in panel (F) for GrzB expression in PD1^+^ CD8^+^ T cells neighboring different tumor cells. Created in BioRender. Kang, Z. (2025) https://BioRender.com/jdn6pqa. **F)**. Violin plot of GrzB expression in PD1^+^ CD8^+^ T cells adjacent to pRPA32^+^ or pRPA32- cells. **G)**. Schematic visualization of immune cell infiltration differences between ATRi responders and non-responders. Graphical components created in BioRender. Kang, Z. (2025) https://BioRender.com/skidcxz.

To explore the spatial tumor microenvironment for biomarker discovery, we performed tissue cyclic multiplex immunofluorescence (t-CycIF) (37) on the corresponding formalin-fixed paraffin embedded (FFPE) tumor tissues using 15 markers cell types and functional states (Fig 4A; STable 6). Image analysis and single-cell quantification identified distinct tumor and immune cell populations including myeloid cells, CD8^+^ T cells and CD8^-^/CD4^+^ T cells. Interestingly, the majority of iPDCs responding to the ATRi combinations were characterized by higher levels of replication stress, indicated by increased pRPA32-RPA2(Ser8) expression in the tumor cells (Fig 4F,G). This finding is in line with previous results from a Phase II clinical trial suggesting that replication stress is a predictor of ATRi response (45). To explore the spatial tumor-immune landscapes, we utilized integrative spatial analyses quantifying the distribution of cell-cell nearest-neighbor distances, and calculates an immune infiltration index quantifying the relative enrichment of immune cells in intratumoral regions compared to stromal regions (SFig 4C; **methods**). We computationally defined the tumor-stroma interface (TSI) and quantified the distribution of cellular distances to the TSI, enabling statistical assessment of immune infiltration patterns both globally and across the TSI (Fig 5A).

Analysis of the iPDC source tumors revealed that tumors responsive to ATRi exhibited a significantly higher intratumoral immune infiltration, mostly driven by the intratumoral PD1^+^ CD8^+^ T cells (Fig 5B; SFig 4C,D). Moreover, immune cells, and specifically the T cells and PD1^+^ CD8^+^ T cells were located significantly closer to the stromal side of the TSI in the responders as compared to the non-responders (Fig 5B; SFig 4E-G). This suggests increased infiltration and aggregation of PD1^+^ CD8^+^ T cells at the TSI in tumors responding to ATRi (Fig 5B; SFig 4E). Interestingly, the overall expression levels of GrzB and PD1 were significantly higher in the immune cells of tumors responding to ATRi (Fig 5C,D).

To assess the spatial associations of tumor cell and intratumoral CD8^+^ T cell states, we next assessed the pRPA32-stratified tumor neighborhoods and PD1^+^ CD8^+^ T cell GrzB expression levels (Fig 5E). Interestingly, we found that GrzB expression was significantly higher in PD1^+^ CD8^+^ T cells proximity of pRPA32^+^ tumor cells as compared to pRPA32^-^ tumor cells (Fig 5F; SFig 4H; SFig 5), suggesting enhanced local activation of CD8^+^ T cells upon spatial proximity to pRPA32^+^ tumor cells.

In summary, our single-cell analysis revealed that ATRi-sensitive tumors were characterized by elevated tumor cell pRPA32 expression (Fig 5G). Further, statistical assessment revealed enhanced peri and intratumoral infiltration of PD1^+^ CD8^+^ T cells. Spatial interactions of intratumoral PD1^+^ CD8^+^ T cells with pRPA32^+^ tumor cells associated with enhanced GrzB activity potentially priming the spatial TME to respond to ATRi. Together, our spatial and functional results suggest a pre-existing intra and peritumoral CD8^+^ T cell - spatial activation phenotype (Fig 5G), which is further augmented in IPDCs upon ATR inhibition in tumors responding to ATR.

## Discussion

Here we established a proof-of-concept patient-derived immunocompetent culture platform of HGSC tumors and validated it using histology, genomic and single-cell as well as spatial analysis. Our results show that iPDCs faithfully recapitulate the source tumors and mirror the clinical treatment responses. Using high-throughput single-cell response profiling, we identify responders to ATR inhibitors alone and in combination with immunotherapeutic agents. Moreover, using spatial analysis we uncover distinct tumor-immune functional crosstalk underpinning responses to ATRi, suggesting a role for T cell activation upon responses to ATRi, and highlighting the potential of our iPDC platform in advancing precision oncology in HGSC.

Tissue-specific matrices have been shown to improve the prediction of treatment responses by preserving the native ECM cues in pancreatic cancer and lymphatic metastases (21,22). Previous studies on HGSC organoid cultures have mostly utilized mouse sarcoma-derived BME matrix (51). Our results demonstrate that the unique OmGel provides an optimal growth environment recapitulating the TME of metastatic HGSC. Previous proteomic analysis confirmed that OmGel consists of key extracellular matrix components closely mimicking human omental tissue, with no detectable levels of cytokines or growth factors in OmGel (23). In line with this, we show that neither the digestion method nor OmGel matrix alters immune cell phenotypes, indicating that all the immune cells from the source tumors are faithfully recapitulated in the iPDCs. As the omentum is the most frequent site of intraperitoneal metastases also in other tumor types (52), our OmGel iPDC model presents as highly relevant for a broad range of gynecological and gastrointestinal cancers.

Thus far, high-throughput drug response profiling in HGSC has focused on monotypic cancer cell cultures or organoids, using compounds that target oncogenic drivers (53). Moreover, while previous studies have mainly involved measurement of metabolic markers such as ATP irrespective of the cell types in the cultures (54), our single-cell image-based readout uniquely captures tumor and immune cell specific functional states. The iPDC responses recapitulate previous findings from clinical trials correlating HRD and PD-1 response (12), as well as replication stress and responses to ATRi (45), and *in vivo* responses in syngeneic mouse models of HGSC (9). Moreover, the iPDC responses were closely aligned with the patients’ clinical responses, highlighting the potential or our platform to identify effective treatment options for HGSC. However, evaluating the clinical relevance of the therapy options presented in our study will require further prospective validation.

ATR inhibitors have been clinically explored in combination with other DNA-damaging agents, such as PARPi and WEE1 inhibitors, in ovarian cancer (51,52). However, the combination of ATR inhibitors with immunotherapy has not been previously investigated. We herein report that combination of ATRi with both investigational (ATXi), and established (anti-PD1 antibody) immunotherapies showed responses in a subset of patients. Furthermore, we found that higher concentrations (1μM or above) of ATRi were toxic to both cancer and immune cells, and lowering concentrations ATRi (0.1μM) in combination with ATXi resulted in cancer cell-specific cytotoxicity without affecting immune cell viability. Tumor cell-death responses to this combination were aligned with CD8^+^ T cell and CD11c^+^ myeloid cell activation. Importantly, our biomarker analysis using single-cell spatial profiling uncovered significantly different T cell infiltration patterns in ATRi responders. Moreover, we identified a link between tumor replication stress signaling and immune activation: CD8^+^ T cells in proximity to pRPA32^+^ tumor cells exhibited significantly elevated GrzB expression. This indicates localized T cell activation neighboring replication stress–high tumor cells and provides evidence that the spatial proximity to distinct tumor phenotypes may prime or enhance cytotoxic immune responses. Altogether, our results are consistent with previous *in vivo* findings, supporting the ATR inhibitor-immunotherapy combination as a promising potential treatment option (9), highlighting the value of our fully humanized iPDC platform in uncovering effective combinatorial treatments and tissue-based predictive biomarkers in HGSC. The spatial analyses show the potential of biomarker discovery combined with functional testing in iPDCs, however we acknowledge that the conclusions will require prospective clinical validation.

The limitations of iPDCs include firstly their short-term culture duration, lasting up to one week, without the ability for passage over time. This constraint is inherent to short-term *ex vivo* models that aim to sustain the viability and functional states of the immune cells. Moreover, we focused our analyses on the most abundant immune cell populations, leaving room for minor cell populations to be further explored. Secondly, this platform only allows for the investigation of immunotherapy effects on intra-tumoral immune cells but does not capture the mobility and priming of peripheral blood immune cells. Thirdly, the limited number of PARPi and chemoresistant patients in our study is a result of the difficulty in obtaining these samples, necessitating further validation with a larger cohort of recurrent samples. Despite these limitations, our iPDC platform harboring patient-matched intratumoral immune cells present a valuable preclinical model for evaluating the effectiveness and biomarkers for new targeted and immuno-oncology agents in HGSC (55).

## Supplementary Material

1

2

3

4

5

6

## Data Availability

The scRNAseq data were produced by the authors, and the read count data was deposited to GEO with the accession number GSE307725. Other data were generated by the authors and are available from the corresponding author upon a reasonable request. Code used for t-CycIF spatial analyses can be found at (https://github.com/farkkilab/Nagaraj-et-al).

## Synopsis

The authors report Ataxia telangiectasia and Rad3-related (ATR) inhibition combined with immunotherapy for high-grade serous ovarian cancer (HGSC) in an immunocompetent ex vivo platform, using histological analyses, drug testing, genomic profiling, single-cell studies, and spatial biomarker discovery.
