# Supplementary material for: Ex Vivo Immuno-Oncology Platform Reveals Spatial T Cell Infiltration Patterns Linked to ATR Inhibition Responses in High-Grade Serous Ovarian Cancer
Source: Cancer Immunol Res. Author manuscript; Available in PMC 2026 Mar 10. (PMC7618831; doi:10.1158/2326-6066.CIR-25-0743)
Supplement: 1 [file EMS212305-supplement-1.pdf]

Supplemental Figure 1

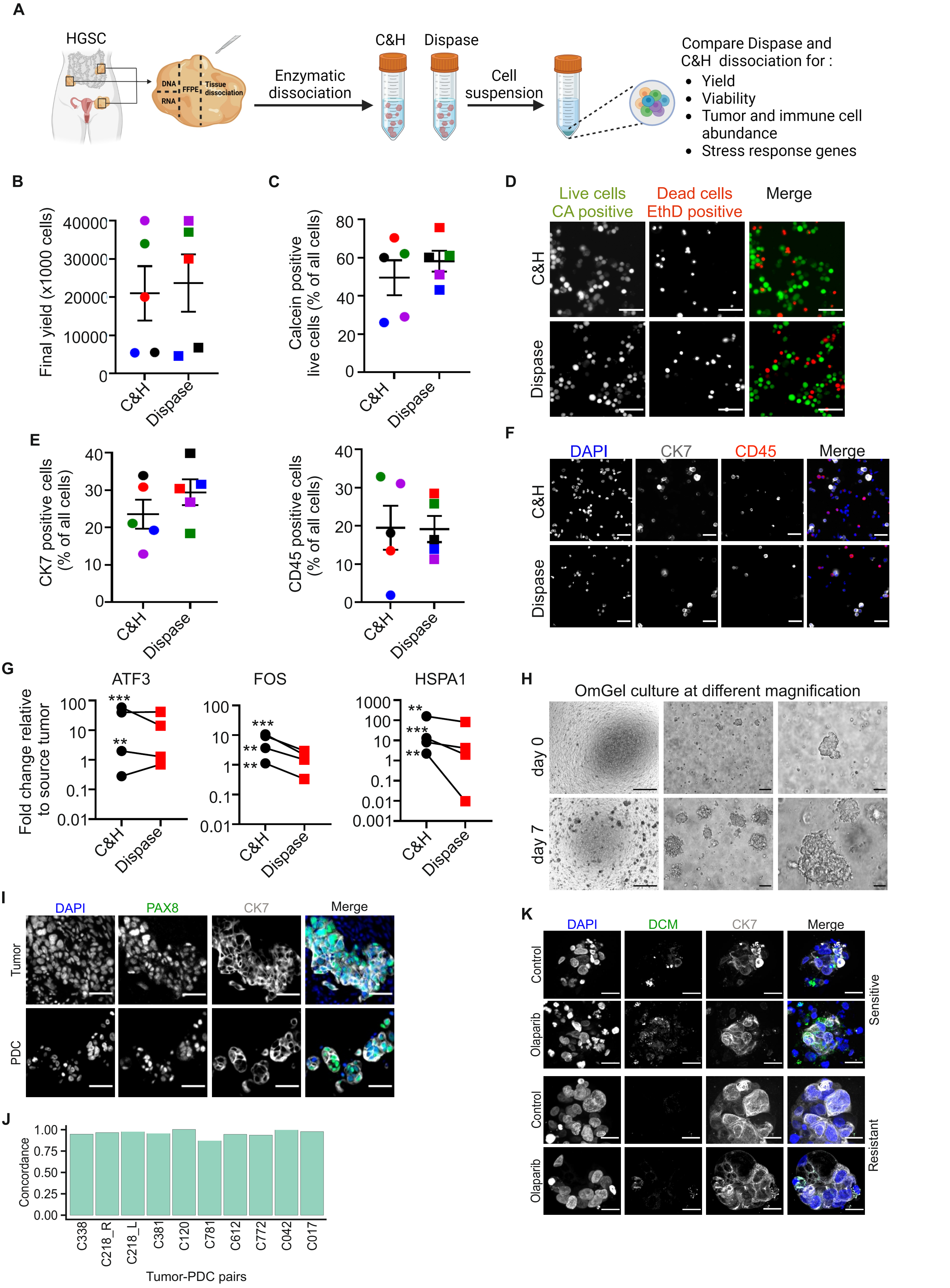

SFig 1. Exploring the iPDCs: enzymatic tissue dissociations' influence on the expression of stress response genes, IF staining and concordance of tissue-specific mutations of source tumor and iPDC. A). Workflow for evaluating the effect of enzymatic dissociation on the final yield, recovery of tumor and immune cells, and expression of stress response genes. Created in BioRender. Nagaraj, A. (2025) https://BioRender.com/lo74hjd B) & C). Quantification of the final yield, and cell viability following C&H or dispase dissociation, each color represents an individual sample. D). Representative IF images showing calcein (CA) positive live cells or Ethidium homodimer-1 (EthD) positive dead cells from C&H or dispase dissociation. Scale bar 50μM. E). Quantification of % of CK7 or CD45 positive cells following C&H or dispase dissociation, each color represents an individual sample. F). Representative IF images of the cells dissociated with C&H or dispase and stained with CK7 or CD45 antibodies. Scale bar 50μM. G). qPCR analysis showing the expression of stress response genes ATF3, FOS, and HSPA1 in the cells dissociated with C&H or dispase. H). Representative brightfield images of d0 and d7 OmGel cultures at different magnifications. Scale bar from left to right: 1mm, 100μM, 50μM. I). IF images of tumor tissue and matched PDCs cultured in OmGel and stained with indicated antibodies. Scale bar 50μM. J). Barplot representing the concordance between tumor tissue and matched PDCs for tissue-specific mutations. K). IF images of control or olaparib-treated PDCs stained with dead cell marker (DCM) and CK7 antibody. Scale bar 50μM.
