# Supplementary material for: Ex Vivo Immuno-Oncology Platform Reveals Spatial T Cell Infiltration Patterns Linked to ATR Inhibition Responses in High-Grade Serous Ovarian Cancer
Source: Cancer Immunol Res. Author manuscript; Available in PMC 2026 Mar 10. (PMC7618831; doi:10.1158/2326-6066.CIR-25-0743)
Supplement: 2 [file EMS212305-supplement-2.pdf]

Supplemental Figure 2

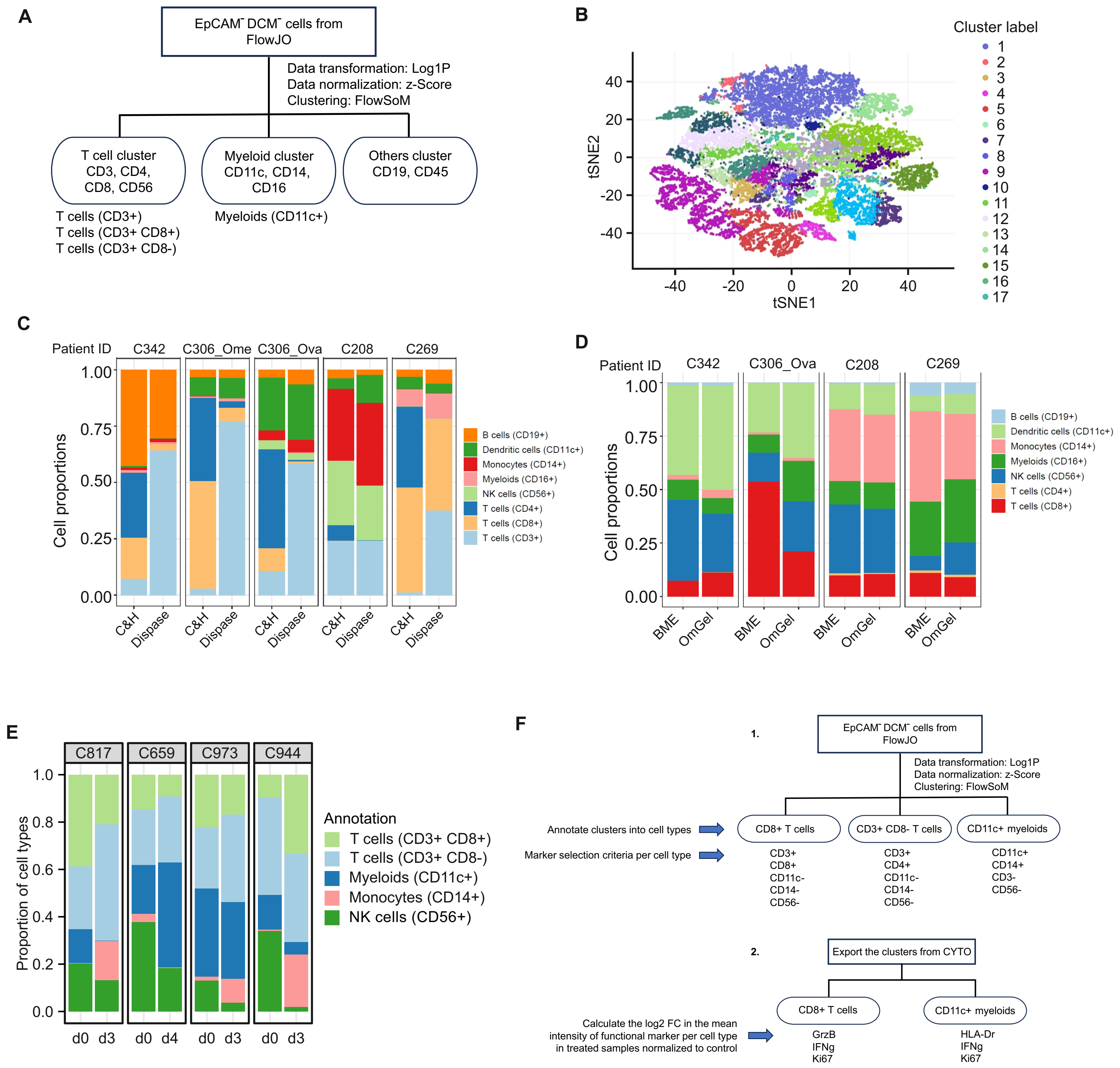

SFig 2. iPDCs maintain source tumor immune cells, showing genotype-specific immune cell responses to Olaparib treatment. A). Flowchart describing the steps involved in flow cytometry data using CYTO. EpCAM- live cells gated from the FlowJo are analyzed in a hierarchical manner to finally annotated into different cell types. B). t-SNE plot depicting the clusters analyzed from EpCAM- cells of tumor and matched iPDCs (n=6), cultured for 4-6 days. C). Bar plot showing the proportions of indicated immune cells following C&H and dispase dissociation. D). Bar plot showing the proportions of indicated immune cells in iPDCs cultured using BME and OmGel for 4-6 days. E). Bar plot showing the proportions of indicated immune cells following three or four days of iPDC culture. F). Flowchart describing the steps involved in flow cytometry data using CYTO. EpCAM- live cells gated from the FlowJo are clustered into different cell types in CYTO. The clusters from CYTO were analyzed for Log2FC in the mean intensity of indicated functional markers per different cell types.
