# Supplementary material for: Ex Vivo Immuno-Oncology Platform Reveals Spatial T Cell Infiltration Patterns Linked to ATR Inhibition Responses in High-Grade Serous Ovarian Cancer
Source: Cancer Immunol Res. Author manuscript; Available in PMC 2026 Mar 10. (PMC7618831; doi:10.1158/2326-6066.CIR-25-0743)
Supplement: 3 [file EMS212305-supplement-3.pdf]

Supplemental Figure 3

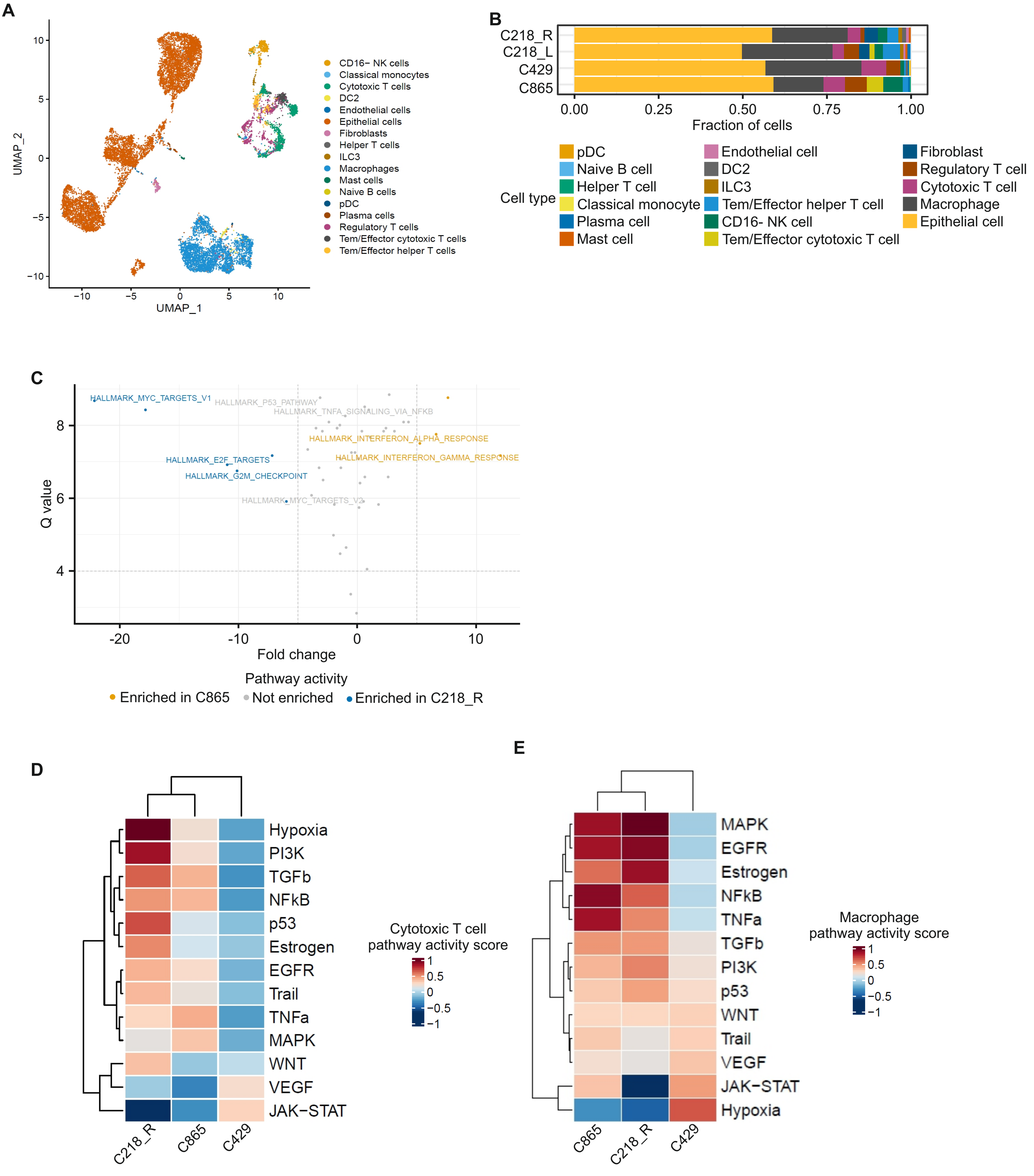

SFig 3. PARPi and chemotherapy resistant tumors show distinct pathway activity across different immune cell types. A). UMAP plot showing epithelial and immune cell type clusters from the tumors of three resistant patients analyzed by scRNAseq. B). Box plot showing fractions of cell types across samples shown in Fig. 3C. C). Scatter plot showing the comparison of pathways enriched in epithelial cells from C865 vs C218_R. D,E). Heatmap showing the pathway activity scores in CD8+ T cells (D) and macrophages (E). from the tumors of resistant patients.
