# Supplementary material for: Ex Vivo Immuno-Oncology Platform Reveals Spatial T Cell Infiltration Patterns Linked to ATR Inhibition Responses in High-Grade Serous Ovarian Cancer
Source: Cancer Immunol Res. Author manuscript; Available in PMC 2026 Mar 10. (PMC7618831; doi:10.1158/2326-6066.CIR-25-0743)
Supplement: 4 [file EMS212305-supplement-4.pdf]

Supplemental Figure 4

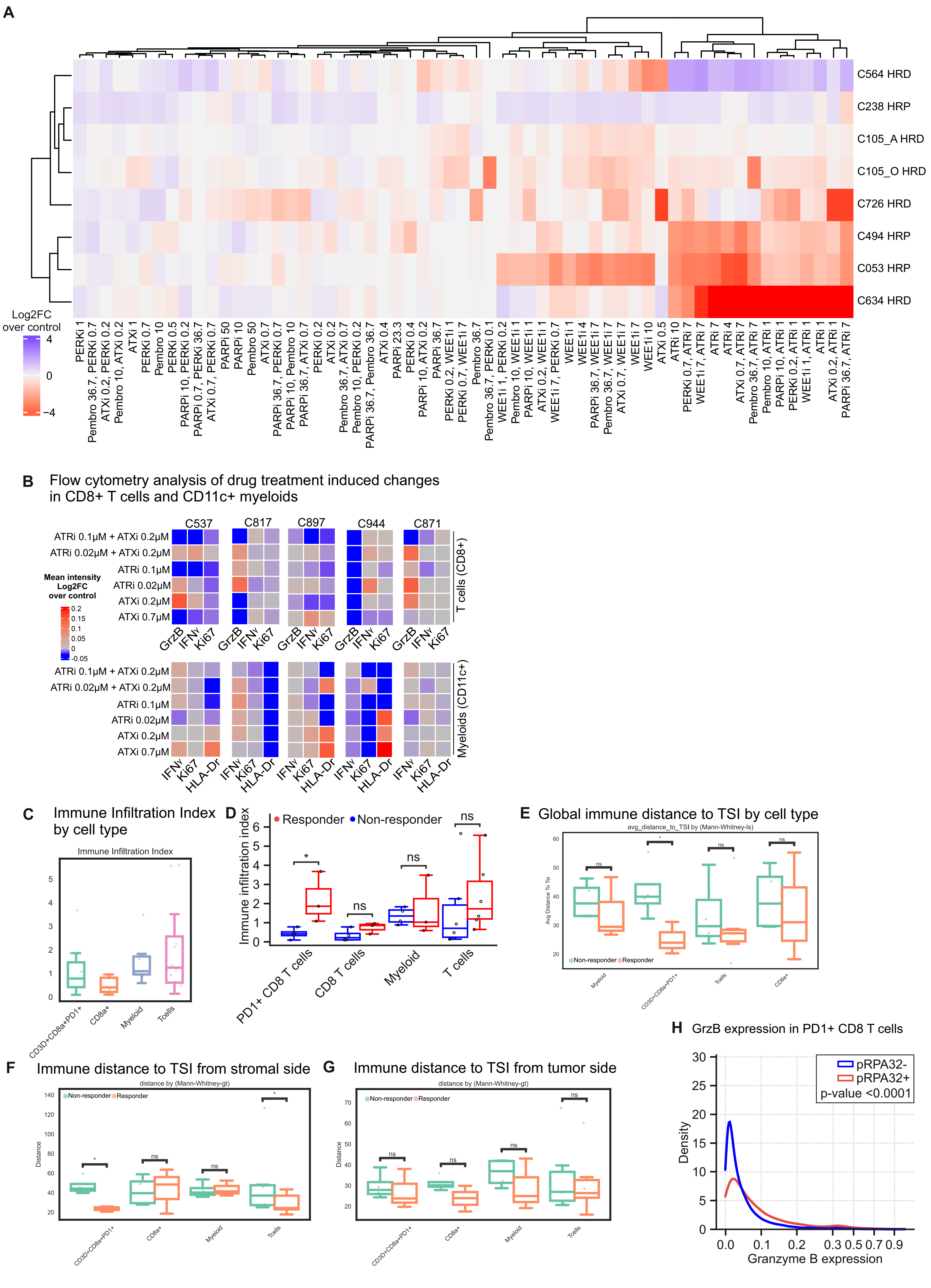

SFig 4. iPDCs provide a platform for high-throughput drug testing. A). Heatmap showing the unsupervised hierarchical clustering of Log2FC in live CK7+ tumor cells over all live cells in each treatment condition normalized to control, across 8 different tumor-derived iPDCs. B). Heatmaps showing the flow cytometry analysis of the indicated markers represented as Log2FC in the mean intensities in CD8+ T cells or CD11c+ myeloid cells upon 3-5 days of treatment with indicated single or combination treatments. The data is normalized to control. C). Immune infiltration index stratified by cell type. D). Immune infiltration index in responders and non-responders, stratified by cell type. E). Global immune cell distance to TSI in responders and non-responders, stratified by cell type. F). Immune cell distance to the tumor-stroma interface (TSI) from the stromal side in ATRi responders vs. non-responders, stratified by immune cell type. G). Immune cell distance to the tumor-stroma interface (TSI) from tumor side in ATRi responders vs. non-responders, stratified by immune cell type. H). Density distributions of GrzB expressing T cells adjacent to pRPA32+ and pRPA32- tumor cells and the expression density is normalized to a scale of 0-1 for improved visualization.
