# Supplementary material for: Ex Vivo Immuno-Oncology Platform Reveals Spatial T Cell Infiltration Patterns Linked to ATR Inhibition Responses in High-Grade Serous Ovarian Cancer
Source: Cancer Immunol Res. Author manuscript; Available in PMC 2026 Mar 10. (PMC7618831; doi:10.1158/2326-6066.CIR-25-0743)
Supplement: 5 [file EMS212305-supplement-5.pdf]

Supplemental Figure 5

A Nearest neighbor distributions

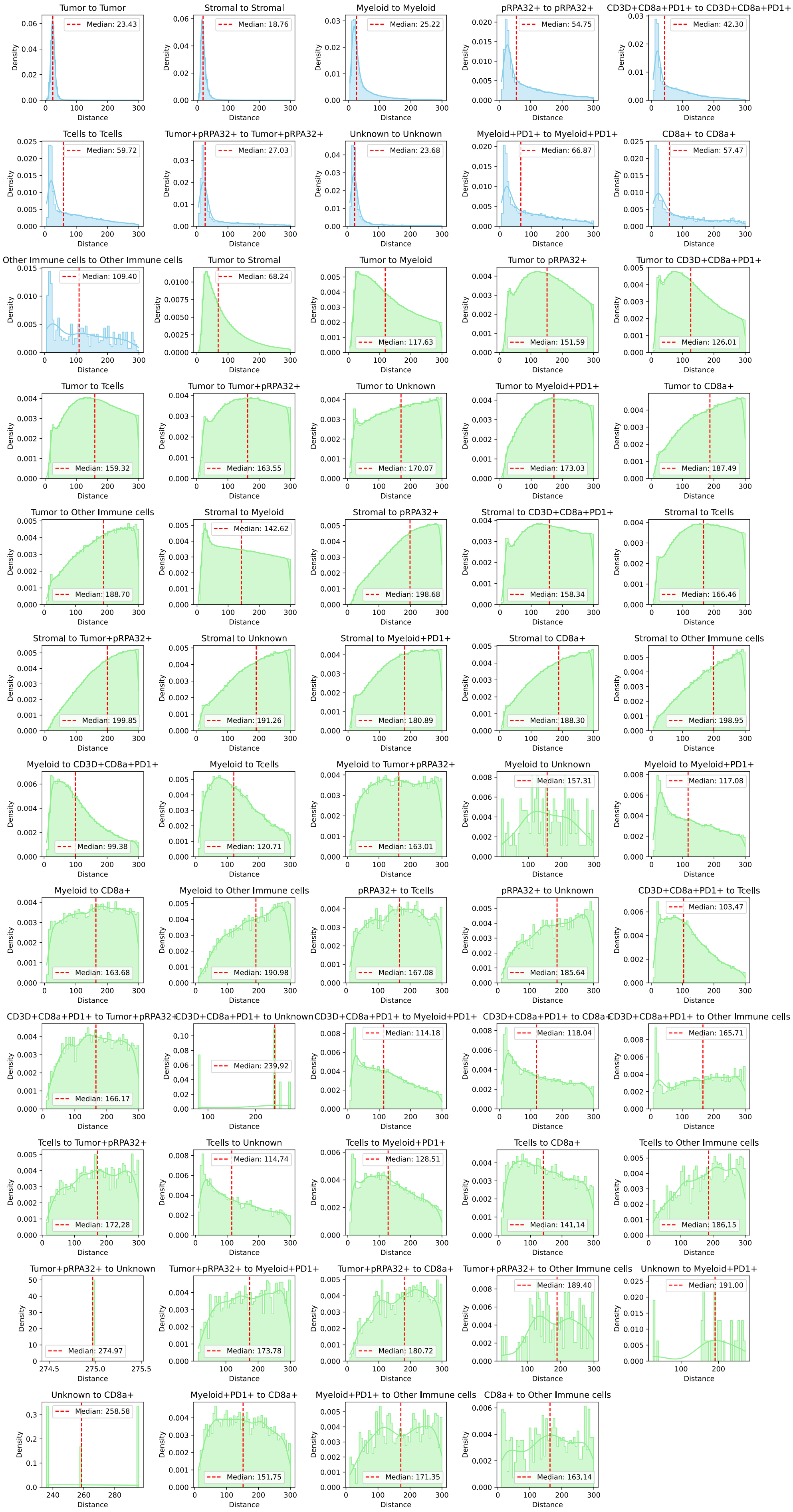

SFig 5. Cell-cell distance overview for t-CycIF validation experiment. A). Histograms showing the cell-cell centroid distance distribution across all cell type pairs. The x-axis is the distance in pixels (1 pixel = 0.325μm) and the y-axis is the density of the distribution curve. Median values are labeled on each subplot.
