# Supplementary material for: Ex Vivo Immuno-Oncology Platform Reveals Spatial T Cell Infiltration Patterns Linked to ATR Inhibition Responses in High-Grade Serous Ovarian Cancer
Source: Cancer Immunol Res. Author manuscript; Available in PMC 2026 Mar 10. (PMC7618831; doi:10.1158/2326-6066.CIR-25-0743)
Supplement: 6 [file EMS212305-supplement-6.docx]

**Supplementary table 1: Primers used for the RT-qPCR analysis.**

| **Gene** | **Forward primer sequence** | **Reverse primer sequence** |
| --- | --- | --- |
| FOS | GACCTATCTGGGTCCTTCTAT | GACGAAGGAAGACGTGTAAG |
| ATF3 | CAAAGTGCCGAAACAAGAAG | CGATGAAGGTTGAGCATGTA |
| HSPA1A | AGGACATCAGCCAGAACA | TGGTGATGGACGTGTAGAA |
| RSP13 | GACGTGAAGGAGCAGATTTAC | CAGGAGCAAGTCCCTTAGA |

**Supplementary table 2: Composition of iPDC growth medium.** Once prepared, medium I was used for up to one month. The medium II supplemented with cytokines is freshly prepared prior to setting up of iPDC cultures.

| **Name** | **Component** | **Stock**  **Concentration** | **Working**  **Concentration** | **Company/**  **Catalogue** |
| --- | --- | --- | --- | --- |
| Medium I | Advanced  DMEM/F12 |  |  | Thermo Scientific/  #12634-010 |
|  | Primocin | 50 mg/ml | 100 μg/ml | Invivogen / #ANTPM1 |
|  | NEAA | 100x | 1x | Gibco/ #11140035 |
|  | Sodium Pyruvate | 100x | 1x | Gibco/ #11360-039 |
|  | L-Glutamine | 100x | 1x | Gibco Life Technologies/  #35050-061 |
|  | HEPES | 100x | 1x | Sigma Merck/ #H0887-100ML |
|  | N-Acetylcysteine | 100 mM | 1 mM | Sigma/ #A9165-100G |
|  | Nicotinamide | 100 mM | 5 mM | Sigma/ #N0636-500G |
|  | Supplement B-27 | 50 x | 1X | ThermoFischer/ #17504044 |
|  | FGF-10 | 100 μg/mL | 10 ng/mL | Peprotech/ #100-26 |
|  | basic FGF (FGF4) | 100 μg/mL | 10 ng/mL | Peprotech/ #AF-100-18B |
|  | A-83-01 | 10 mM | 0.5 μM | Sigma/ #SML0788-5MG |
|  | β-estradiol | 10 mM | 10 nM | Sigma/ #E2758-1G |
|  | Neuregulin-1 /  Heregulin | 6.6 uM /  50 ug/ mL | 5 nM | Peprotech/ #100-03 |
|  | EGF | 100 μg/mL | 5 ng/mL | Peprotech/ #AF-100-15 |
|  | Hydrocortisone | 1 mg/mL | 500 ng/mL | Sigma/ #H0888 |
|  | Y-27632 /  ROCK inhibitor | 5 mM | 5 μM | AbMole Biosciences/  #M1817 |
| Medium II  (Medium I + cytokines) | IL2 | 66 X E6 IU/Ml | 500 IU/mL | Peprotech/ #200-02 |
|  | IL15 | 10 μg/ml | 10 ng/ml | Peprotech/ #200-15 |
|  | IL7 | 10 μg/ml | 10 ng/ml | Peprotech/ #200-07 |

**Supplementary table 3: Flow cytometry gating strategies**

**
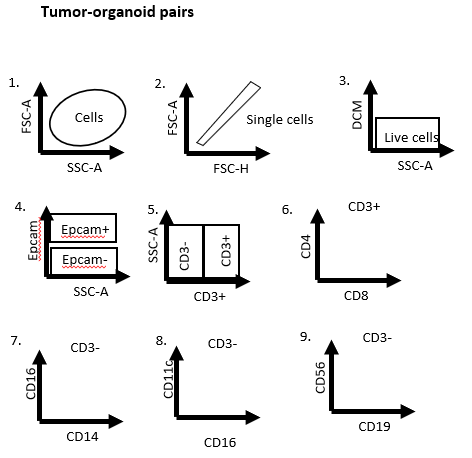
**

**
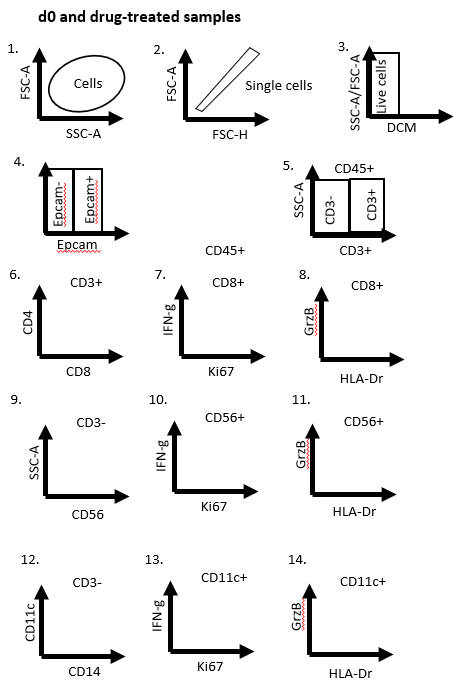
**

**Supplementary table 4: Details of compounds used for single agent treatments**

| **Compound** | **Catalog number** | **Company** | **Solvent** | **Working concentrations** |
| --- | --- | --- | --- | --- |
| Olaparib | S1060 | Selleckchem | DMSO | 10 µM |
|  |  |  |  | 23 µM |
|  |  |  |  | 37 µM |
|  |  |  |  | 50 µM |
| Berzosertib (VE-822) | S7102 | Selleckchem | DMSO | 1 µM |
|  |  |  |  | 4 µM |
|  |  |  |  | 7 µM |
|  |  |  |  | 10 µM |
| Adavosertib | S1525 | Selleckchem | DMSO | 1 µM |
|  |  |  |  | 4 µM |
|  |  |  |  | 7 µM |
|  |  |  |  | 10 µM |
| Ziritaxestat (GLPG1690) | HY-101772 | MedChemExpress | DMSO | 0.2 µM |
|  |  |  |  | 0.5 µM |
|  |  |  |  | 0.7 µM |
|  |  |  |  | 1 µM |
| AMG PERK 44 | HY-12661A | MedChemExpress | DMSO | 0.2 µM |
|  |  |  |  | 0.5 µM |
|  |  |  |  | 0.7 µM |
|  |  |  |  | 1 µM |
| Pembrolizumab | A2005 | Selleckchem | PBS | 10 mg/mL |
|  |  |  |  | 23 mg/mL |
|  |  |  |  | 37 mg/mL |
|  |  |  |  | 50 mg/mL |

**Supplementary table 5: Details of compound combinations for combinatorial treatments**

| **Combination** | **Compound** | **Concentration** |
| --- | --- | --- |
| 1 | Olaparib + Pembrolizumab | 10 μM + 10 μg/ml |
| 2 | Olaparib + Pembrolizumab | 36 μM + 36 μg/ml |
| 3 | Olaparib + Ziritaxestat (GLPG1690) | 10 μM + 0.2 μm |
| 4 | Olaparib + Ziritaxestat (GLPG1690) | 36 μM + 0.7 μm |
| 5 | Olaparib + AMG PERK 44 | 10 μM + 0.2 μm |
| 6 | Olaparib + AMG PERK 44 | 36 μM + 0.7 μm |
| 7 | Olaparib + Berzosertib (VE-822) | 10 μM + 1 μM |
| 8 | Olaparib + Berzosertib (VE-822) | 36 μM + 7 μM |
| 9 | Olaparib + Adavosertib | 10 μM + 1 μm |
| 10 | Olaparib + Adavosertib | 36 μM + 7 μm |
| 11 | Berzosertib (VE-822) + Ziritaxestat (GLPG1690) | 1 μM + 0.2 μm |
| 12 | Berzosertib (VE-822) + Ziritaxestat (GLPG1690) | 10 μM + 0.7 μm |
| 13 | Berzosertib (VE-822) + AMG PERK 44 | 1 μM + 0.2 μm |
| 14 | Berzosertib (VE-822) + AMG PERK 44 | 10 μM + 0.7 μm |
| 15 | Berzosertib (VE-822) + Adavosertib | 1 μM + 1 μm |
| 16 | Berzosertib (VE-822) + Adavosertib | 7 μM + 7 μm |
| 17 | Adavosertib + Ziritaxestat (GLPG1690) | 1 μm + 0.2 μm |
| 18 | Adavosertib + Ziritaxestat (GLPG1690) | 7 μm + 0.7 μm |
| 19 | Adavosertib + AMG PERK 44 | 1 μm + 0.2 μm |
| 20 | Adavosertib + AMG PERK 44 | 7 μm + 0.7 μm |
| 21 | Adavosertib + Pembrolizumab | 1 μm + 10 μg/ml |
| 22 | Adavosertib + Pembrolizumab | 7 μm + 36 μg/ml |
| 23 | Pembrolizumab + Ziritaxestat (GLPG1690) | 10 μg/ml + 0.2 μm |
| 24 | Pembrolizumab + Ziritaxestat (GLPG1690) | 36 μg/ml + 0.7 μm |
| 25 | Pembrolizumab + AMG PERK 44 | 10 μg/ml + 0.2 μm |
| 26 | Pembrolizumab + AMG PERK 44 | 36 μg/ml + 0.7 μm |
| 27 | Pembrolizumab + AMG PERK 44 | 10 μg/ml + 0.2 μm |
| 28 | Pembrolizumab + AMG PERK 44 | 36 μg/ml + 0.7 μm |
| 29 | Ziritaxestat (GLPG1690) + AMG PERK 44 | 0.2 μm + 0.2 μm |
| 30 | Ziritaxestat (GLPG1690) + AMG PERK 44 | 0.7 μm + 0.7 μm |

**Supplementary table 6: scRNAseq QC metrics**

| **Sample** | **Sequencing depth** | **Number of reads** | **% on target reads** | **% Reads mapping to reference** | **Number of cells** | **Median UMI counts per cell** | **Median genes per cell** |
| --- | --- | --- | --- | --- | --- | --- | --- |
| C685 | 53743 | 376954909 | 0.581 | 0.953 | 7014 | 6125 | 1522 |
| C429 | 47148 | 390055069 | 0.619 | 0.966 | 8273 | 6233 | 1950 |
| C218_L | 79330 | 282255377 | 0.557 | 0.972 | 3558 | 6850 | 1694 |
| C218_R | 89370 | 320659975 | 0.593 | 0.983 | 3588 | 8263 | 1846 |

**Supplementary table 7: Antibodies used in t-CycIF panel.**

| **Antibody** | **Fluorochrome** | **Company** | **Cat number (RRID)** | **Clone** | **Dilution** |
| --- | --- | --- | --- | --- | --- |
| Hoechst | 361 | Invitrogen | H3570 | *blank* | 10000 |
| Granzyme B | unconj (647) | Dako | M7235 (AB_2114697) | GrB-7 | 50 |
| CD68 | 488 | CST | 24850S (AB_2798886) | D4B9C | 100 |
| Phospho-Stat1 (Y701) | 555 | CST | 8183S (AB_10860600) | 58D6 | 100 |
| CD8a | 660 | Invitrogen | 50-0008-80 (AB_2574148) | AMC908 | 150 |
| Vimentin | 555 | CST | 9855S (AB_10859896) | D21H3 | 100 |
| Phospho-RPA32/RPA2(Ser8) | 488 | CST | 31912S | E5A2F | 100 |
| CD3D | 555 | Abcam | ab208514 (AB_2728789) | EP4426 | 100 |
| PD1 | 647 | Abcam | ab201825 (AB_2728811) | EPR4877(2) | 150 |
| CD4 | 488 | RnD systems | FAB8165G (AB_2728839) | polyclonal | 150 |
| CD11C | 555 | CST | 77882BC (AB_3331656) | D3V1E | 150 |
| Pax8 | 488 | Abcam | ab214955 (AB_3713022) | EPR18715 | 150 |
| FoxP3 | 555 | Invitrogen | 41-4777-80 (AB_2573608) | 236A/E7 | 100 |
| HLA-DPB1 | 647 | Abcam | ab201347 (AB_2861375) | EPR11226 | 400 |
| CK7 | 555 | Abcam | ab209601 (AB_2728790) | EPR17078 | 200 |
| gH2Ax | 647 | BioLegend | 613407 (AB_2114994) | 2F3 | 150 |
